# Physiological fidelity or model parsimony? The relative performance of reverse-toxicokinetic modeling approaches

**DOI:** 10.1186/s12918-017-0407-3

**Published:** 2017-03-11

**Authors:** Michael A. Rowland, Edward J. Perkins, Michael L. Mayo

**Affiliations:** 1 Oak Ridge Institute for Science and Education, Oak Ridge, TN USA; 2 Environmental Laboratory, US Army Engineer Research and Development Center, Vicksburg, MS USA

## Abstract

**Background:**

Physiologically-based toxicokinetic (PBTK) models are often developed to facilitate in vitro to in vivo extrapolation (IVIVE) using a top-down, compartmental approach, favoring architectural simplicity over physiological fidelity despite the lack of general guidelines relating model design to dynamical predictions. Here we explore the impact of design choice (high vs. low fidelity) on chemical distribution throughout an animal’s organ system.

**Results:**

We contrast transient dynamics and steady states of three previously proposed PBTK models of varying complexity in response to chemical exposure. The steady states for each model were determined analytically to predict exposure conditions from tissue measurements. Steady state whole-body concentrations differ between models, despite identical environmental conditions, which originates from varying levels of physiological fidelity captured by the models. These differences affect the relative predictive accuracy of the inverted models used in exposure reconstruction to link effects-based exposure data with whole-organism response thresholds obtained from in vitro assay measurements.

**Conclusions:**

Our results demonstrate how disregarding physiological fideltiy in favor of simpler models affects the internal dynamics and steady state estimates for chemical accumulation within tissues, which, in turn, poses significant challenges for the exposure reconstruction efforts that underlie many IVIVE methods. Developing standardized systems-level models for ecological organisms would not only ensure predictive consistency among future modeling studies, but also ensure pragmatic extrapolation of in vivo effects from in vitro data or modeling exposure-response relationships.

**Electronic supplementary material:**

The online version of this article (doi:10.1186/s12918-017-0407-3) contains supplementary material, which is available to authorized users.

## Background

There has been a strong push to determine the hazards of chemicals to public health using high-throughput in vitro assays, which has the benefit of being more relevant, fast, inexpensive, and more humane than reliance on animal models. However, in vitro data may not accurately reflect in vivo exposure concentrations due to absorption, distribution, metabolism, and excretion (ADME) of the chemical throughout the body [1]. Physiologically based toxicokinetic (PBTK) modeling has been recognized as a key tool for in vitro to in vivo extrapolation (IVIVE): PBTK models simulate the distribution and accumulation of a chemical throughout various tissues after exposure on an in vivo or whole animal scale [2–6]. These models have traditionally been designed using a top-down approach; that is, the model structure is usually chosen and parameterized according to the simplest design that best fits the apical effect data, often collapsing the physiology of the organism to include only a handful of connected compartments [1, 5–11]. To our knowledge, there have been no systematic studies investigating the relationship between the physiological fidelity of PBTK model architecture and the accuracy and precision of their predictions for chemical uptake and disposition throughout an organism.

One technique related to PBTK modeling is reverse toxicokinetics (rTK, also known as reverse dosimetry or exposure reconstruction). A primary goal of rTK modeling is to provide a predictive platform that can be used to determine the minimum chemical concentration to which an organism must be exposed to achieve an internal steady state observed to cause an effect at a particular organ [12]. An important issue facing reverse toxicokinetics is that TK models may be “ill-posed”, meaning they may not be uniquely invertible or the solution’s behavior may not be continuous [13, 14]. To address this problem, rTK modeling often involves a Bayesian approach to exposure reconstruction using Markov Chain Monte Carlo simulations of a “forward” PBTK model to infer likely exposure concentrations [5, 10, 14–19]. The availability and quality of biomonitoring data and the approximate nature of Monte Carlo simulations, which result in a probability distribution rather than a single estimate, often limits the accuracy of estimates from probabilistic rTK models [14].

Here we investigate how PBTK model complexity affects the fidelity of its dynamical predictions, and how this comparison can be improved by relaxing constraints on the modeled kinetics. For each PBTK model, we make two assumptions in addition to those inherent to the modeling approach that serve to simplify our analyses: 1) environmental exposure concentrations are constant; and 2) chemical metabolism and excretion rates can be modeled using first order mass-action kinetics. These assumptions allow for an exact, unique analytical solution for the predicted exposure concentration (PEC) as a function of whole-body or tissue concentrations for each PBTK model considered here. Despite that each model considered here conceptualizes the same animal system — an aquatic vertebrate, a teleost fish — exposure concentrations predicted from our rTK models in this manner varied by approximately 2–3 orders of magnitude. To understand how model design affects its predictions, we characterize how the architecture of each model affects the dynamics of bioaccumulation and tissue clearance (relaxation of the concentration profiles to equilibrium) in tissues of both individual organs and the whole body. We find that if chemical exposure is constant, then steady state whole body and tissue chemical concentrations vary substantially between models, dependent upon the identity of the chemical stressor. These differences are further evidenced by examining the relaxation half-lives, a measure of a chemical’s residence time within a certain tissue or clearance from the whole-body. We further observe that relaxation times vary greatly between models, with different chemicals changing only the values associated with an individual model.

These results indicate that choosing how closely the PBTK model design matches the animal physiology is critical for achieving accurate predictions in modeling studies in which parameter values are identified separately from available in vitro assay data. PBTK modeling is becoming a tool used by the toxicology, pharmacology, and systems biology communities for risk and hazard assessment, drug discovery, and basic research into chronic diseases [9, 11, 20–22]. We therefore argue for standardization of the design, training, and validation of PBTK and their associated rTK models in future modeling studies. While this would ensure predictive consistency across computational studies, it would also guarantee that conclusions and recommendations could be compared fairly when evaluating different health-related outcomes.

## Methods

The systems of ordinary differential equations for each model and the derivations of the associated rTK models are written explicitly in the Supplemental Information. Ordinary differential equations were numerically integrated using CVODE from SUNDIALS, using a dense linear solver with the backward differentiation formula and a Newton iteration methodology [23]. Steady states were chosen heuristically by sight: the long time series data were initially obtained for the PBTK models, and time to reach steady state was chosen visually. Statistical tests were performed in R [24].

## Results

### Reverse toxicokinetics for in vitro to in vivo extrapolation: a proof of concept

Is it possible to use reverse toxicokinetic modeling as a predictive framework for IVIVE? We approached this question by evaluating the fidelity of each model for predicting an external chemical-dependent lowest observable effect concentration (LOEC), defined as the lowest experimental exposure level observed to elicit an adverse effect in a whole animal, calculated using only in vitro concentration-response data subject to very few constraints. This semi-quantitative approach should be viewed with high uncertainty, as we compare the results of one type and scale (i.e., in vitro data of molecular-scale effects) against many different experiments of another type and scale (i.e., whole-organism toxicity measurements). Ideally, we would seek data from individual experiments that report both whole-animal exposure and accumulated concentrations in tissues. However, to be clear, our goal is to first investigate and then to place bounds on the efficacy of associating high-throughput in vitro results with exposure data; as such, the aforementioned rigorous approach is outside the scope of our present effort.

To proceed, we first obtained concentrations at which 10% of the maximum response activity is observed from an assay, labeled AC10, for a set of endocrine-disrupting chemicals with estrogenic activity from the dataset published by Judson, et al. [25]. Given our focus on estrogenic chemicals, we restricted our analyses to in vitro assays testing for the activity of estrogen receptor alpha (ERα). The AC10 is a reasonable measure of the concentration for which the in vitro assay activity departs from control levels, which can be seen by first expanding a sigmoid concentration-response function about its inflection point to first-order in the semi-log scale, and then computing the intersection of this line with the control levels. The concentration coincident with this intersection corresponds to a response of approximately 12%, which can be understood as the value for which the response crosses into the power-law regime of activity from the control regime (See Additional file 1, section 8 for derivation). While a more rigorous approach would be to use biological or technical replicates to estimate the concentration of the first statistically significant point-of-departure from control (POD) on an assay-by-assay basis, the AC10 serves as a compromise favoring the availability of high-throughput measurements and use in determining preliminary or screening level exposure values. As a final input into the reverse toxicokinetic models, we obtained LogP values for these estrogenic chemicals [25, 26], which we considered reasonable estimates for octanol-water partition ratios.

Whole-organism level data for each of the estrogenic chemicals were collated from LOECs reported for disruption of the reproductive systems of fish from the ECOTOX database [27]. These LOECs are derived entirely from experimental studies and therefore depend strongly on the experimental design, which varied across the data sourced from ECOTOX. We should note that the LOEC measurements we obtained from ECOTOX should be treated as approximations for POD concentrations at the whole-organism level, because of their reliance on a limited range of tested exposure concentrations. Moreover, this variance between POD and LOEC cannot be reasonably quantified unless the authors have employed more sophisticated estimates for the POD. In the future, POD concentrations would be preferable and may be calculated if enough time series replicates were available. The scope of this work, however, is comparing the performance of the models rather than assessing their accuracy for deployment for IVIVE modeling, and, as such, the LOEC values provide an acceptable approximation.

**Fig. 1:**
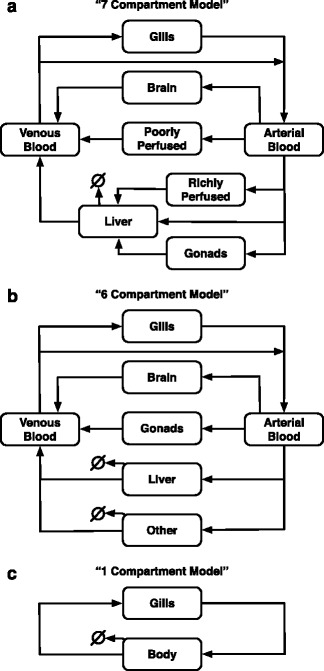
Diagrams of the different PBTK models. **a** The “7-Compartment” model, which is based off of a published PBTK model for zebrafish (*Danio rerio*) [6]. The gills are assumed to be constantly at steady state with the environmental chemical concentration. Chemical in the gills can then flow into the body, entering first into the arterial blood. The arterial blood flows directly into the brain, gonads, liver, poorly perfused tissue, and richly perfused tissue. Chemical exiting the gonads and richly perfused tissue flows directly into the liver. The liver is the only organ that can metabolize the chemical. Chemical exiting the brain, liver, and poorly perfused tissue enters the venous blood, which can either exit the body through the gills or gets recycled into the arterial blood. **b** The “6-Compartment” model, which is based off a published PBTK model for fathead minnow (*Pimephales promelas*) [5]. Chemical in the environment enters the arterial blood through the gills and is then distributed to the brain, gonads, liver, and “other” tissues. Both the liver and “other” tissues can metabolize and excrete the chemical, respectively. Chemical exiting these four compartments enter the venous blood and either exit the body through the gills or re-enters the arterial blood. **c** The “1-Compartment” model, representing a simplification of a fish to a single compartment with gills. Here, chemical in the environment enters the body through the gills. Some of the chemical can then be metabolized, and whatever is not retained in the body can exit through the gills

We developed, or adapted from the literature, three PBTK models (Fig. 1): two multicompartment models based individually on the physiology of the teleosts zebrafish (*Danio rerio*) and fathead minnow (*Pimephales promelas*), and a third that conceptualizes these systems as a single whole-body compartment. The first model, a “7-Compartment model” (7C), is based on the PBTK model for zebrafish developed by Pery et al. [6]. This 7C model conceptualizes arterial and venous blood, brain, gonads, liver, poorly and richly perfused tissues into 7 distinct compartments. The 7C model mimics the connected structure of the zebrafish model, and employs the same parameterization. The second model, a “6-Compartment model” (6C), which is structurally based on a systems biology model of the hypothalamic-pituitary-gonadal axis in female fathead minnow developed by Li et al. [5]. This 6-compartment model includes arterial and venous blood, brain, gonads, liver, and “other” tissues, but does not distinguish between highly and poorly perfused tissues, as in the 7C model. The 6C model adopts the model structure of the fathead minnow TK model, but employs body-centric parameter values from the 7C model in cases where their interpretation is analogous (e.g., water-flow rate through gills). Finally, the third model is a “1-Compartment model” (1C), an exemplary PBTK model in which the fish is represented by having gills (which serve as uptake/elimination pathways) and a single compartment representing the rest of the body. The 1C model has precedent in previously published human pharmacokinetic models, wherein the human body is abstracted as a well-mixed single compartment chemical reactor [28, 29]. The associated rTK models were derived by assuming each of the models to be at steady state, then inverting the systems mathematically, obtaining expressions for the exposure concentration as functions of bodily concentrations (See Additional file 1, sections 3 and 6 for more detail).

As mentioned previously, we, in order to derive an exact analytical solution for the chemical exposure concentration as a function of the whole body or organ-specific concentrations, make two basic assumptions: 1) environmental exposure concentrations are constant; and 2) chemical metabolism and excretion rates can be modeled using first order mass-action kinetics. We compared the 7C PBTK model using both first order mass-action kinetics and Michaelis kinetics for the metabolic and excretion rates to determine if our assumption would have an appreciable effect on the dynamics. We found no evidence supporting a difference in the dynamics of the two models (See Additional file 1, section 4 for more detail).

**Fig. 2:**
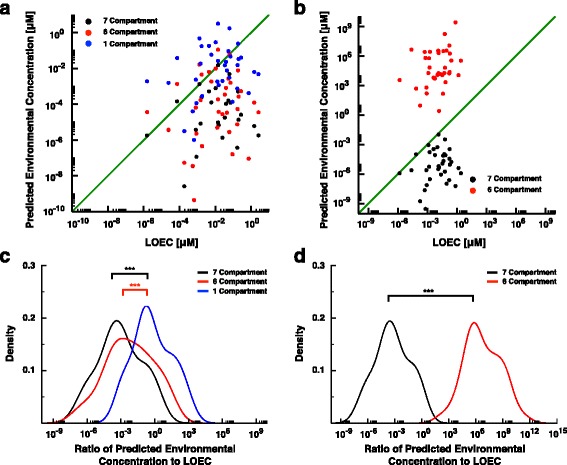
Influence of model design on reverse toxicokinetic predictions for EDCs with estrogenic activity (**a**) The predicted environmental concentration of EEDCs from the 7C (*black dots*), 6C (*red dots*) and 1C (*blue dots*) models vs. the experimentally determined LOEC. For the 1C model, only 21 of the 36 predicted concentrations are less than the experimentally-determined LOEC (*p* = 0.405, binomial test). The 6C and 7C model predictions are often lower than the LOECs (30/36, *p* = 6.96 × 10^−5^ and 32/36, *p* = 1.942 × 10^−6^, respectively). **b** The predicted environmental concentration of EEDCs from the 7C (*black dots*) and 6C (*red dots*) models vs. the experimentally determined LOEC. The published AC10 concentrations were, in this case, assigned to be the steady state chemical concentrations in the gonads and the environmental concentrations predicted solely from the gonad compartments. 35 of 36 predictions were less than the experimental LOEC in the 7C model (*p* = 1.077 × 10^−9^, binomial test) while all 36 predictions were higher than the experimental LOEC in the 6C model (*p* = 2.911 × 10^−11^, binomial test). **c** The kernel density estimates of the ratio of the predicted environmental concentrations from the rTK models to the associated LOEC for the EEDCs, using the published AC10 values as whole body concentrations. The 6C (*red*) and 7C rTK models (*black*) produce predicted environmental concentrations whose ratios to the respective LOECs are significantly smaller than the predictions from the 1C rTK models (*blue*) (*p* = 4.1 × 10^−5^ and 5.785 × 10^−9^, respectively, Student’s *t*-test). The kernel density estimates were obtained in R [24]. **d** The kernel density estimates of the ratio of the predicted environmental concentrations from the rTK models to the associated LOEC for the EEDCs, using the published AC10 values as the chemical concentrations in gonads. The 6C (*red*) and 7C (*black*) rTK models produce predicted environmental concentrations whose ratios to the respective LOECs are significantly different (*p* < 2.2 × 10^−16^, Student’s *t*-test)

We employed AC10 values obtained from high-throughput in vitro assays for the estrogenic chemicals (discussed above) as whole-body, steady-state concentrations in each of our rTK models. The 6C and 7C models generally provide PECs lower than the experimental LOECs for the estrogenic chemicals (Fig. 2a, black (*p* = 6.96 × 10^−5^) and red dots (*p* = 1.942 × 10^−6^), respectively, binomial test). On average, the 1C model predictions provide PECs closer to the LOECs, both over- and under-predicting the ECOTOX LOECs in roughly equal amounts (Fig. 2c, blue dots, *p* = 0.405, binomial test). The relative differences between PECs and LOECs become apparent by taking their ratio: the 6C and 7C models predict environmental concentrations that are far smaller than the 1C predictions (*p* = 4.1 × 10^−5^ and 5.785 × 10^−9^, respectively, Student’s *t*-test). There is, however, no significant difference between the 6C and 7C results (*p* = 0.0802, Student’s *t*-test). Note that comparison with LOECs is used only to provide context for PECs; therefore, our results should not strictly be used to evaluate the absolute accuracy of any individual model. A minimum requirement for understanding the predictive accuracy of these models requires that exposure and tissue measurement data originate from the same experiments. Unfortunately too many whole-organism exposure studies have not measured tissue residues (although, more commonly body burden is measured), and obtaining these experimental endpoints from individual experiments should be a focus in the field moving forward.

One concern with these results is that the AC10s described above are measured in vitro, so it may not be a fair comparison to employ them as body burdens for exposure reconstruction purposes. Because reproductive endpoints for fish are often regulated by estrogen interactions with ERα in hepatoctyes (such as with vitellogenin production) and steroidogenesis takes place in the gonads [30], we alternatively considered the AC10s as gonad concentrations for the 6C and 7C models. Consistent with results from the body-burden implementation (described above), the 7C PECs were mostly smaller in value than associated LOECs (Fig. 2*b*, black dots, *p* = 1.08 × 10^−9^, binomial test). In contrast, the 6C PECs are consistently larger than associated LOECs (Fig. 2b, red dots, *p* = 2.911 × 10^−11^, binomial test). Taking the ratio of the PEC to its associated LOEC, we find that there is a very significant difference between the tissue-specific environmental concentration predictions of the 6C and 7C models (Fig. 2d, *p* < 2.2 × 10^−16^, Student’s *t*-test). The difference in environmental concentration predictions between the models likely arise due to the impacts of model architecture on the dynamics and steady states of the respective PBTK simulations.

### Effects of model complexity on dynamics

**Fig. 3:**
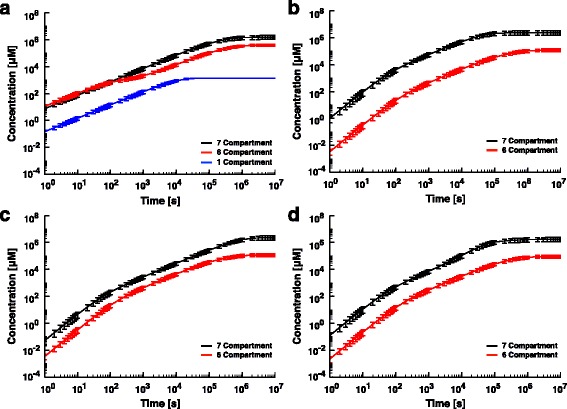
Time series bodily concentrations after initial exposure to an environment with a constant concentration of chemical. **a** The full body concentration of chemical as a function of time. The black, red, and blue curves represent the average accumulation of chemical throughout the body in the 7C, 6C, and 1C models, respectively. The error bars represent the standard deviation in the concentrations at each time point from *n* = 10^3^ simulations. For each simulation we varied the body parameters tested in the sensitivity tests over a log-normal distribution with a mean equal to the literature-derived values. **b** The chemical concentration in the brain as a function of time for the 6C (*red*) and 7C (*black*) models. **c** The chemical concentration in the gonads as a function of time for the 6C (*red*) and 7C (*black*) models. **d** The chemical concentration in the liver as a function of time for the 6C (*red*) and 7C (*black*) models. Note that the small error bars in these figures are likely resultant from the models being generally insensitive to the sampling of the parameter values from log_2_ distributions, with the model demonstrating variances only up to about 10^−1^ (See Additional file 1, section 5). While larger error bars could be obtained by sampling parameter values from a wider distribution, our main concern is the average behavior of the time series given the sensitivity of the model

Although the compartmentalized 6C and 7C models exhibit similar sensitivities in their steady state organ concentrations of the accumulated chemical to similar parameter values, this observation does not take into account any potential differences in the transient dynamics between models. To evaluate potential dynamical variability between the models, we executed 1000 simulations for each one, wherein the body specific parameters were allowed to vary by random sampling from a log-normal distribution. This allows for the inference of time-dependent statistical properties associated with a population of fish, which provides additional insight over the potentially accurate but imprecise deterministic time-series used to model an individual’s dynamical response. In each simulation, the models were again exposed to 10 μM of diazinon, and tissue concentrations were allowed to evolve in time until steady state (Fig. 3).

Initially, the full body concentrations of diazinon in the 6C and 7C models are very similar (Fig. 3a). However, beyond several minutes of simulation, the 6C model’s full body concentration temporarily rises at a rate lower than observed for 7C, resulting in a smaller steady-state value. Interestingly, the 1C model’s full body concentration rises at a rate similar to that of the 7C, but shows significant delay. For instance, the full-body concentration of the 7C model achieved by 1 min requires approximately 1 h of simulation time for the 1C model. Additionally, the 1C reaches a significantly lower steady-state value sooner than the other models (~10^4^ s vs. ~10^6^ s).

Similarities between the whole body concentration dynamics of the 6C and 7C models mask differences in chemical concentrations accumulated in organ compartments represented in both models. The concentrations of diazinon in the brain, gonads, and liver in the 6-compartment model is consistently about 1–2 orders of magnitude less than the concentrations in the brain, gonads, and liver in the 7C (Fig. 3b-d). This suggests that observing only the whole body concentration, or the chemical concentration in a single tissue may overlook more complex behaviors in other tissues. The differences in the 6C and 7C model time series, however, do seem to be dependent upon the identity of the chemical stressor. We therefore repeated these simulations with 1,2-dicholoroethane, which has a lower octanol-water ratio (log K_ow_ = 1.48) than diazinon (log K_ow_ = 3.81). The concentration of 1,2-dichloroethane in the brain, gonads and liver over time after initial exposure showed a higher degree of overlap between the 6C and 7C models compared against the diazinon exposures, and the 6C model’s full body concentrations exceeded that of the 7C (Additional file 1: Figure S1).

### Effects of model complexity on relaxation

**Fig. 4:**
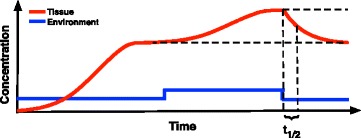
Diagram illustrating the computational simulation to find the relaxation time for the model. The environmental concentration is kept at a constant value until the concentration in all tissues reaches a steady state. The environmental concentration is then increased slightly and kept at the new level until the tissues reach the new steady state. The environmental concentration is then reduced back to its original level. We then measured the amount of time it took for the whole body and for each tissue to drop halfway back to its original steady state value (the relaxation half-life, *t*_*1/2*_)

Given differences in model dynamics reported in earlier sections, both in whole body and tissue-specific compartments, we further examined how each model responded to minor perturbations to the chemical concentration in the environment. First, and for each model, tissue concentrations were allowed to reach a steady state when exposed to a constant chemical concentration. Next, the exposure concentration was slightly increased and the model was allowed to achieve a new steady state. The environmental concentration was then reset to its original concentration, and we measured the amount of time needed for the whole body and tissue-specific concentrations to relax halfway back to their original steady state values, the relaxation half-life (Fig. 4). For each model this procedure was repeated for 1000 simulations in which body parameters were varied randomly as described in the previous section.

**Fig. 5:**
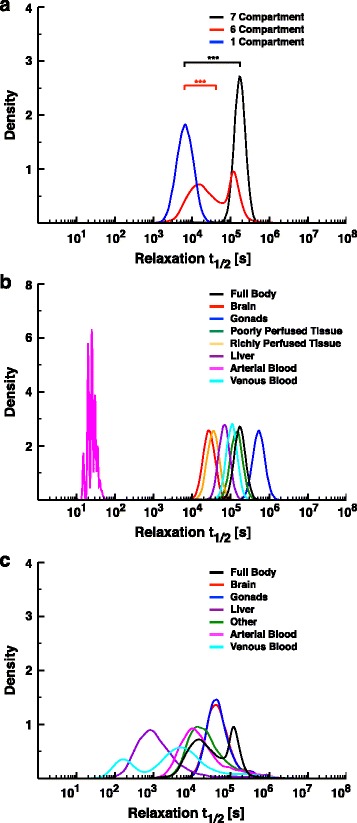
Relaxation dynamics of the PBTK models. **a** The kernel density plot of the relaxation half-lives of the whole-body chemical concentrations in the 7C (*black*), 6C (*red*), and 1C (*blue*) models. The sample populations of half-lives were taken from *n* = 10^3^ simulations varying the body parameters in a fashion similar to that described for Fig. 4. The differences in the means from the 7C vs. 1C and 6C vs. 1C are statistically significant (*p* < 0.01, Student’s *t*-test). Note that the relaxation half-lives are plotted on a log_10_ scale. **b** The kernel density plot of the relaxation half-lives of the whole body (*black*) and tissue-specific chemical concentrations for the brain (*red*), gonads (*blue*), poorly perfused tissue (*green*), richly perfused tissue (*orange*), liver (*purple*), arterial blood (magenta), and venous blood (cyan). Note that the relaxation half-lives are plotted on a log_10_ scale. **c** The kernel density plot of the relaxation half-lives of the whole body (black) and tissue-specific chemical concentrations for the brain (*red*), gonads (*blue*), liver (*purple*), “other” tissues (*green*), arterial blood (*magenta*), and venous blood (*cyan*). Note that the relaxation half-lives are plotted on a log_10_ scale

The whole body relaxation half-lives of the three models varied over several orders of magnitude. The 7C model exhibited the slowest recovery, requiring at least an order of magnitude more time, on average, than either of the other two models (Fig. 5a). The most rapid recovery was observed from the 1C model, which required, on average, less than 10^4^ s (~2.8 h). The 6C and 7C models show a wide range of relaxation half-lives within specific tissues (Fig. 5b & c).

The gonads in the 7C model, on average, exhibit half-lives over an order of magnitude larger than observed from any other tissue or that of the full body relaxation (Fig. 5b, blue) suggesting that toxicants may accumulate and persist in the gonads much longer than the rest of the body. Therefore gonads may provide a favorable target for testing chemical concentrations in fish. On the one hand, gonad tissues would be affected less by fluctuations in exposure concentrations over longer periods of time; on the other hand, the arterial blood is quick to relax with a half-life on the order of seconds (Fig. 5b, magenta), and suggests that bioavailability (the fraction of unchanged chemical found in the blood) may not be a useful endpoint through which to correlate dynamics of acute small fish toxicity.

The 6C tissues show a wider degree of variation in relaxation half-lives than those in the 7C model. For example, across simulations, the venous blood’s relaxation half-life varied from around 10^2^ s (c. 2 min) to nearly 10^6^ s (c. 11 days; Fig. 5c, cyan). In contrast to the 7C model, none of the 6C tissues stood out as having relaxation half-lives distinct from the others. To check if these results were specific to diazinon, we also ran the relaxation simulations with 1,2-dichloroethane (Additional file 1: Figure S2). The more hydrophilic chemical shortened relaxation half-lives for the full body concentrations in all three models. However, the general result of the 1C model being the fastest to relax of the three and the 7C model being slowest held (Additional file 1: Figure S2B & C). An observed shift to faster relaxation half-lives held for the tissues in both the 7C and 6C models. Additionally, variances in the 6C tissues shrunk considerably, providing differences in compartment-specific relaxation dynamics comparable to that seen in the 7C model. Sensitivity analyses on the impacts of variations in the magnitude of individual body parameters for each model reveal that the relaxation half-lives for whole body and tissue-specific chemical concentrations generally depends on the respiratory flow rate, blood flow rate, and total mass of the organism in each model (Additional file 1: Figure S3). Note that these sensitivity analyses demonstrate a relative robustness of the different compartments to many of the body parameters in the 7C model, a behavior not seen in the 6C or 1C models. We hypothesize that such a difference in robustness may arise from then inclusion of an assimilation factor in the expressions for the exchange of chemical from the environment into the blood and the converse in the 7C model, a parameter missing from the other two, or from differences in model structure. Intuitively, a more hydrophilic chemical should be easier to clear different organs, as it is less likely to reside longer in lipid bilayers, and smaller organisms with faster flow rates would be able to more rapidly expel chemical from organs than a larger organism with a slower flow rate.

### Effect of model architecture on reverse toxicokinetic predictions

We have found that differences in model architecture for aqueous organisms can be associated with differences in transient dynamics and relaxation to steady state in response to changes in stressor concentration. This begs the question: How do differences in PBTK model structure and response in aqueous organisms affect the PECs obtained from associated rTK models?

**Fig. 6:**
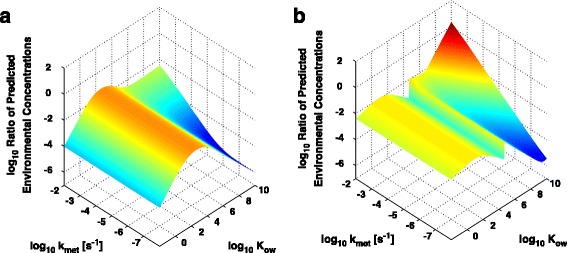
Influence of model design on reverse toxicokinetic predictions. **a** The log_10_ of the ratio of the predicted environmental concentrations of the 6C rTK model vs. the 1C model as a function of the log_10_ metabolism/excretion rate (k_met_) and the log_10_ octanol-water ratio (K_ow_). Note that for the observed range of values for k_met_ and K_ow_ we see no points in which the 6C model overestimates the 1C model (log_10_ ratio of concentrations > 0). **b** The log_10_ of the ratio of the predicted environmental concentrations of the 7C rTK model vs. the 1C model as a function of the log_10_ metabolism rate (k_met_) and the log_10_ octanol-water ratio (K_ow_). Note that the 7C model only overestimates the 1C model (log_10_ ratio of concentrations > 0) at high values of k_met_ and K_ow_

The rTK models require three common chemical-dependent parameters as input: a hepatic clearance rate (k_met_); the octanol-water ratio (K_ow_); and a dissociation constant of chemical with binding proteins in plasma. Due to an absence of experimental data to characterize the binding of chemical with blood components, we have assumed that both arterial and venous blood do not accumulate chemicals. This assumption could alter the concentration of the chemical within the body. However, as the binding mechanism would be consistent between the 6C and 7C models it should not affect the relative accuracy, only altering the absolute magnitude of the predictions of the models. We evaluated the models over a range of values for the hepatic clearance rate and the octanol-water ratio with a whole-body chemical concentration of 10 μM, to understand how these two independent parameters generally affected the exposure predictions independent of any specific chemical. Results of these comparisons are visualized in Fig. 6, and illustrate the ratio of modeled PECs (6C vs. 1C and 7C vs. 1C, respectively) as a function of these parameters. At low values of K_ow_ (low lipid solubility), metabolism/excretion rates had no appreciable effect on the ratio of predictions for neither the 6C nor 7C; however, this ratio was more strongly affected at K_ow_ values greater than approximately 10^5^ (high lipid solubility), which is intuitive because chemical must partition into the tissues before metabolism can act to destroy/transform it, therefore eliminating it from tissue. Additionally, between the two models, only the 7C was shown to over-estimate compared to the 1C model (ratio > 1) within the range of values for these parameters tested; the ratio rose above 1 for improbably high K_ow_ and metabolism/excretion rates (Fig. 6b).

To understand how the model predictions diverge from one another based solely on model structure without the impact of parameterization, we developed two additional PBTK models based on the 6C model. In the first, the gonads and other tissue compartments are collapsed into a single “other” tissue compartment. In the second, the gonads have been made to empty into the liver directly rather than into the venous blood, as is seen in the 7C model. We solved for the steady state chemical concentrations in each of the compartments as functions of the exposure concentration. We then calculated the difference in concentrations for the arterial blood, venous blood, brain, and liver between models using the analytical expressions. Collapsing the gonads and other tissues into a single compartment had no effect on the steady state chemical concentrations in the other compartments. However, the steady states of these compartments in the model with the gonads emptying into the liver rather than the venous blood were greater than those of the original model, regardless of the values chosen for the parameters (See Additional file 1, Section 7 for derivations and details). The reverse toxicokinetic model associated with the rewired version thus consistently under-estimates the environmental concentration with respect to the original model. This behavior is similar to that of the 7C model, which has two compartments emptying into the liver, compared to the 6C and 1C models, where all tissue compartments empty into the venous blood. These results demonstrate that the architecture of the model can have a significant impact on the relative accuracy and precision of the rTK models for aqueous exposure reconstruction.

## Discussion

In this work we aimed to characterize how PBTK model structure can affect the dynamics of the model and the relative accuracy of rTK environmental concentration predictions based on different model structures. We found that the more complex 6C and 7C models exhibited faster dynamics, reaching particular whole body concentrations sooner than the 1C model. Additionally, the 6C and 7C models also reached steady state whole body concentrations orders of magnitude higher than the 1C model. This behavior depended upon the chemical being simulated: chemicals with larger octanol-water ratios ended up displaying higher bodily concentrations in the 7C model, while the difference is minimized or reversed for chemicals with low octanol-water ratios. The differences in model dynamics extended to responses to perturbations in exposure concentration while at steady state. The whole body concentration of the 7C model took longer to relax to a new steady state, while the 1C model was the fastest, regardless of the nature of the chemical. We also found that focusing on whole body relaxation overlooked the differences in relaxation between different compartments within the 7C model. The arterial blood achieved its new steady state on the order of minutes, whereas most of the body took on the order of hours to days to relax. However, relaxation in gonads took longer than the rest of the body, suggesting that they may contain toxicants far longer than other tissues; such accumulation would be missed without direct testing of gonad tissues. This hypothesis that gonad tissues retain chemical for much longer time periods could be tested by extending the sampling design of long-term aqueous experimental studies.

The differences in dynamics between the models also affected the relative accuracy of the associated rTK models. We found that for biologically- and chemically-relevant metabolism/excretion rates and chemical octanol-water ratios, the 1C model consistently over-estimated the minimum exposure concentrations based on similar whole body concentrations compared to either the 6C or 7C models. These results highlight the need to further study and understand the influence of model architecture on rTK accuracy.

One potential issue suggested by our results involves parameterization. Wherever applicable, we used similar parameter values for each model (e.g., the same lipid and water content for the brain in both the 6C and 7C models). However, there remained a set of parameters for each model taken from literature values based on values in different species (i.e., zebrafish for the 7C model-specific parameters and fathead minnow for 6C). This is a common concern in PBTK and rTK modeling, as most investigations develop and parameterize a new model on a case-by-case basis [2, 5, 6, 10, 14, 16–19, 31]. While differences in parameterization likely contributes to variation in dynamics and steady states between models, our analytical study demonstrates that the model structure also impacts steady state predictions for aqueous organisms. In the future it would be of interest to systematically analyze the influence of parameterization and chemicals-specific properties on the relative and absolute accuracy of PBTK and rTK models. While the current study focuses on contrasting relative performance of predictions from different models, a detailed analysis of their absolute accuracy is necessary to develop guidelines for standardizing the design and parameterization practices associated with PBTK and rTK model development for aquatic organisms.

Increasing the reliability and consistency of in vivo to in vitro extrapolation is crucial to many fields. Historically, IVIVE has been used in determining toxicant or drug distribution and impacts throughout the body using inexpensive, faster, and more humane cell-based studies [32]. There has been, however, a greater appreciation of the difficulties of in vivo to in vitro extrapolations in other fields, including in relation to insulin resistance and cancer. In a review of the application of systems biology in disease studies, Somvanshi and Venkatesh present a systematic map of insulin resistance and defective metabolic homeostasis, including the flow of small molecules and hormones between various tissues. The authors indicate that molecular-level networks present within the cells of each tissue are, in fact, a part of a larger network [11]. A recent computational study found that a three-component model of VEGF transport and kinetics in tumor-bearing mice, built in a top-down approach and with parameters fitted to in vivo data, significantly underpredicts VEGF secretion rates in the tumor compared to reported in vitro rates [9].

## Conclusions

Ultimately, our work demonstrates the influence of PBTK/rTK model structure on relative performance in aqueous organisms with the example of comparing three models based on teleost fish, each with unique model architectures. This demonstrates the tradeoffs in model design and accuracy, pointing out potential impacts on predicted concentrations when favoring parsimony over physiological fidelity, or vice-versa. Our results highlight the need to develop standardized model design and validation procedures to accurately and precisely predict environmental and physiological conditions that lead to measured small molecule concentrations in the body. Standard modeling practices and training and validation data sets would ensure consistency among future computational studies and allow the fair comparison of results across projects studying physiologically similar organisms.

## Additional file

Additional file 1:
**Supplemental Information.** Additional information including supporting figures, the generalized conceptual model for an organ with metabolism, detailed descriptions of the toxicokinetic models, a comparison of the 7C model to the original Zebrafish PBTK on which it was based, results of sensitivity analyses of the PBTK models, detailed descriptions of the reverse toxicokinetic models, an analytical approach to determining the influence of architecture on steady state concentrations, and an approximation of the departure of in vitro data from control levels. (PDF 1762 kb)

## Abbreviations

7C: 7-Compartment
6C: 6-Compartment
1C: 1-Compartment
PBTK: Physiologically-based toxicokinetic
rTK: Reverse toxicokinetic
TK: Toxicokinetic
IVIVE: In vitro to in vivo extrapolation
PECs: Predicted environmental concentration
LOEC: Lowest observed effect concentration
AC10: Concentration at 10% of maximum activity
ADME: Absorption, distribution, metabolism, excretion
ERα: Estrogen receptor α
POD: Point of departure

## References

[1] Rostami-Hodjegan A (2012). Physiologically based pharmacokinetics joined with in vitro-in vivo extrapolation of ADME: a marriage under the arch of systems pharmacology. Clin Pharmacol Ther.

[2] Andersen ME (2003). Toxicokinetic modeling and its applications in chemical risk assessment. Toxicol Lett.

[3] Chen W-Y (2016). Toxicokinetic modeling challenges for aquativ nanotoxicology. Front Mar Sci.

[4] Coecke S, Pelkonen O, Leite SB, Bernauer U, Bessems JG, Bois FY, Gundert-Remy U, Loizou G, Testai E, Zaldivar JM (2013). Toxicokinetics as a key to the integrated toxicity risk assessment based primarily on non-animal approaches. Toxicol In Vitro.

[5] Li Z, Kroll KJ, Jensen KM, Villeneuve DL, Ankley GT, Brian JV, Sepulveda MS, Orlando EF, Lazorchak JM, Kostich M (2011). A computational model of the hypothalamic: pituitary: gonadal axis in female fathead minnows (Pimephales promelas) exposed to 17alpha-ethynylestradiol and 17beta-trenbolone. BMC Syst Biol.

[6] Pery AR, Devillers J, Brochot C, Mombelli E, Palluel O, Piccini B, Brion F, Beaudouin R (2014). A physiologically based toxicokinetic model for the zebrafish Danio rerio. Environ Sci Technol.

[7] Dahl SG, Aarons L, Gundert-Remy U, Karlsson MO, Schneider YJ, Steimer JL, Troconiz IF (2010). Incorporating physiological and biochemical mechanisms into pharmacokinetic-pharmacodynamic models: a conceptual framework. Basic Clin Pharmacol Toxicol.

[8] D'Souza RWaF, WR. and Bruce, RD. and Andersen, ME. Physiologically based pharmacokinetic model for ethylene dichloride and its application in risk assessment. In: Drinking water and health, Volume 8: Pharmacokinetics in risk assessment. Washington: National Academies Press; 1987: pp. 286–311.

[9] Finley SD, Dhar M, Popel AS (2013). Compartment model predicts VEGF secretion and investigates the effects of VEGF trap in tumor-bearing mice. Front Oncol.

[10] Jonsson F, Bois F, Johanson G (2001). Physiologically based pharmacokinetic modeling of inhalation exposure of humans to dichloromethane during moderate to heavy exercise. Toxicol Sci.

[11] Somvanshi PR, Venkatesh KV (2014). A conceptual review on systems biology in health and diseases: from biological networks to modern therapeutics. Syst Synth Biol.

[12] Kanno J, Inoue T (2002). Reverse toxicology as a future predictive toxicology. Toxicogenomics.

[13] Hadamard J (1902). Sur les problèmes aux dérivées partielles et leur signification physique. Bull Univ Princeton.

[14] Lyons MA, Yang RS, Mayeno AN, Reisfeld B (2008). Computational toxicology of chloroform: reverse dosimetry using Bayesian inference, Markov chain Monte Carlo simulation, and human biomonitoring data. Environ Health Perspect.

[15] Bernillon P, Bois FY (2000). Statistical issues in toxicokinetic modeling: a bayesian perspective. Environ Health Perspect.

[16] Covington TR, Robinan Gentry P, Van Landingham CB, Andersen ME, Kester JE, Clewell HJ (2007). The use of Markov chain Monte Carlo uncertainty analysis to support a public health goal for perchloroethylene. Regul Toxicol Pharmacol.

[17] Hack CE, Chiu WA, Jay Zhao Q, Clewell HJ (2006). Bayesian population analysis of a harmonized physiologically based pharmacokinetic model of trichloroethylene and its metabolites. Regul Toxicol Pharmacol.

[18] Kinch CD, Ibhazehiebo K, Jeong JH, Habibi HR, Kurrasch DM (2015). Low-dose exposure to bisphenol A and replacement bisphenol S induces precocious hypothalamic neurogenesis in embryonic zebrafish. Proc Natl Acad Sci U S A.

[19] Allen BC, Hack CE, Clewell HJ (2007). Use of Markov chain Monte Carlo analysis with a physiologically-based pharmacokinetic model of methylmercury to estimate exposures in US women of childbearing age. Risk Anal.

[20] National Research Council (1994). Science and judgment in risk assessment.

[21] US Environmental Protection Agency (2005). Guidelines for Carcinogen Risk Assessment. EPA/630/P-03/001F.

[22] Theil FP, Guentert TW, Haddad S, Poulin P (2003). Utility of physiologically based pharmacokinetic models to drug development and rational drug discovery candidate selection. Toxicol Lett.

[23] Hindmarsh AC, Brown PN, Grant KE, Lee SL, Serban R, Shumaker DE, Woodward CS (2005). SUNDIALS: suite of nonlinear and differential/algebraic equation solvers. ACM Trans Math Softw.

[24] R Core Team: R (2015). A language and environment for statistical computing.

[25] Judson RS, Magpantay FM, Chickarmane V, Haskell C, Tania N, Taylor J, Xia M, Huang R, Rotroff DM, Filer DL (2015). Integrated model of chemical perturbations of a biological pathway using 18 in vitro high-throughput screening assays for the estrogen receptor. Toxicol Sci.

[26] Ghose AK, Viswanadhan VN, Wendoloski JJ (1998). Prediction of hydrophobic (Lipophilic) properties of small organic molecules using fragmental methods: an analysis of ALOGP and CLOGP methods. J Phys Chem A.

[27] US. Environmental Protection Agency (2016). ECOTOX user guide: ECOTOXicology database system. version 4.0.

[28] Rotroff DM, Wetmore BA, Dix DJ, Ferguson SS, Clewell HJ, Houck KA, Lecluyse EL, Andersen ME, Judson RS, Smith CM (2010). Incorporating human dosimetry and exposure into high-throughput in vitro toxicity screening. Toxicol Sci.

[29] Judson RS, Kavlock RJ, Setzer RW, Hubal EA, Martin MT, Knudsen TB, Houck KA, Thomas RS, Wetmore BA, Dix DJ (2011). Estimating toxicity-related biological pathway altering doses for high-throughput chemical risk assessment. Chem Res Toxicol.

[30] Cabas I, Chaves-Pozo E, Garcia-Alcazar A, Meseguer J, Mulero V, Garcia-Ayala A (2013). The effect of 17alpha-ethynylestradiol on steroidogenesis and gonadal cytokine gene expression is related to the reproductive stage in marine hermaphrodite fish. Mar Drugs.

[31] Peters SA (2011). Physiologically based pharmacokinetic (PBPK) modeling and simulations : principles, methods, and applications in the pharmaceutical industry.

[32] Krewski D, Andersen ME, Mantus E, Zeise L (2009). Toxicity testing in the 21st century: implications for human health risk assessment. Risk Anal.

