# Supplementary material for: Physiological fidelity or model parsimony? The relative performance of reverse-toxicokinetic modeling approaches
Source: BMC Syst Biol. 2017 Mar 11;11:35. doi: 10.1186/s12918-017-0407-3 (PMC5346271; doi:10.1186/s12918-017-0407-3)
Supplement: Additional file 1: — Supplemental Information. Additional information including supporting figures, the generalized conceptual model for an organ with metabolism, detailed descriptions of the toxicokinetic models, a comparison of the 7C model to the original Zebrafish PBTK on which it was based, results of sensitivity analyses of the PBTK models, detailed descriptions of the reverse toxicokinetic models, an analytical approach to determining the influence of architecture on steady state concentrations, and an approximation of the departure of in vitro data from control levels. (PDF 1762 kb) [file 12918_2017_407_MOESM1_ESM.pdf]

Physiological fidelity or model parsimony? The relative performance of reverse-toxicokinetic modeling approaches

Supplemental Information

Michael A. Rowland[1,2], Edward J. Perkins[2], and Michael L. Mayo[2]

[1] Oak Ridge Institute for Science and Education, Oak Ridge, TN, USA

[2] Environmental Laboratories, U.S. Army Corps of Engineers, Halls Ferry Road, Vicksburg, MS, USA

Email: michael.l.mayo@usace.army.mil;

Contents

1 Supporting Figures **2**

2 Generalized Conceptual Model for an Organ with Metabolism **5**

3 Toxicokinetic model equations **8**

3.1 7-Compartment PBTK model equations 8

3.2 6-Compartment PBTK model equations 13

3.3 1-Compartment PBTK model equations 16

4 Comparison of the 7-Compartment PBTK to the Zebrafish PBTK model **17**

5 Sensitivity of PBTK models to parameters **20**

6 Reverse toxicokinetic model equations **22**

6.1 7-compartment rTK model equations 22

6.2 6-compartment rTK model equations 25

6.3 1-Compartment rTK model equations 28

7 Influence of Architecture on Steady State Compartment Concentrations **29**

8 Approximation of the departure of *in vitro* data from control levels **35**

1 Supporting Figures

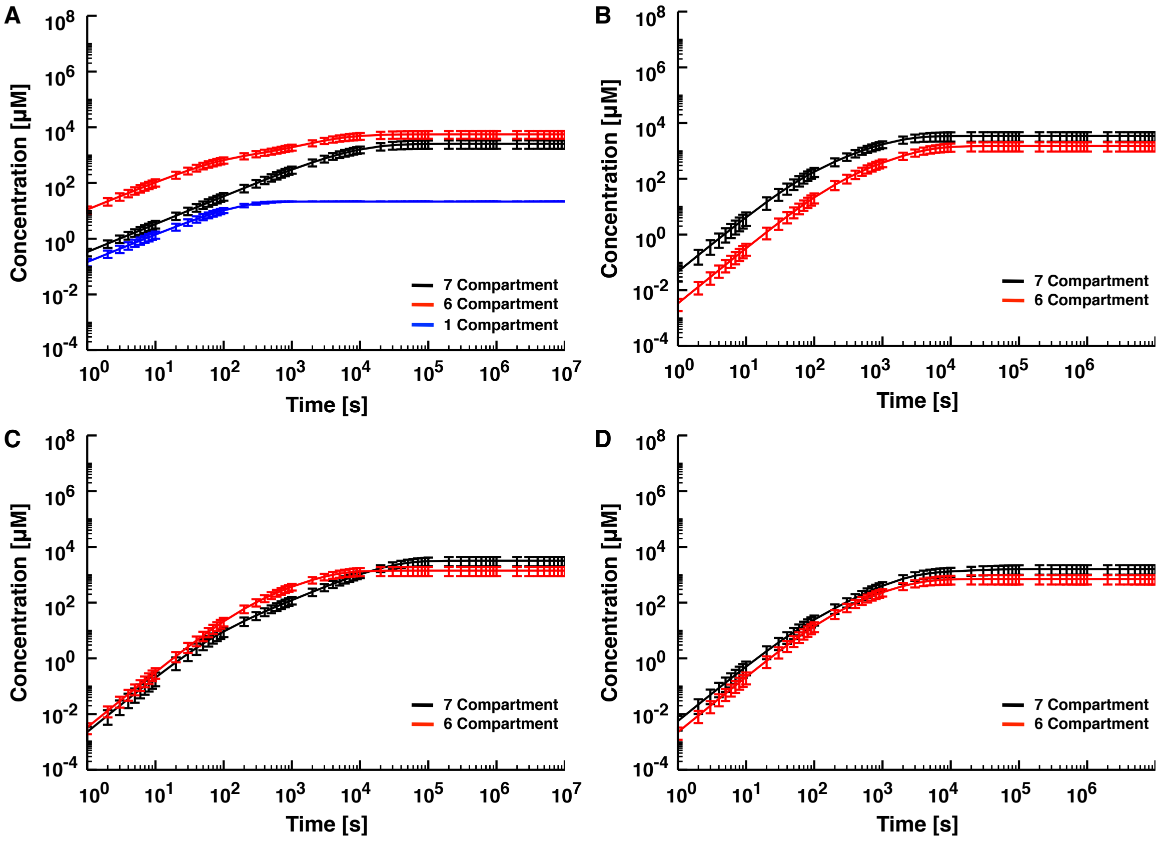

Figure S1: Time series bodily concentrations after initial exposure to an environment with a constant concentration of 1,2-dichloroethane. (A) The full body concentration of chemical as a function of time. The black, red, and blue curves represent the average accumulation of chemical throughout the body in the 7C, 6C, and 1C models, respectively. The error bars represent the standard deviation in the concentrations at each time point from n=103 simulations. For each simulation we varied the body parameters tested in the sensitivity tests over a log-normal distribution with a mean equal to the literature-derived values. (B) The chemical concentration in the brain as a function of time for the 6C (red) and 7C (black) models. (C) The chemical concentration in the gonads as a function of time for the 6C (red) and 7C (black) models. (D) The chemical concentration in the liver as a function of time for the 6C (red) and 7C (black) models.

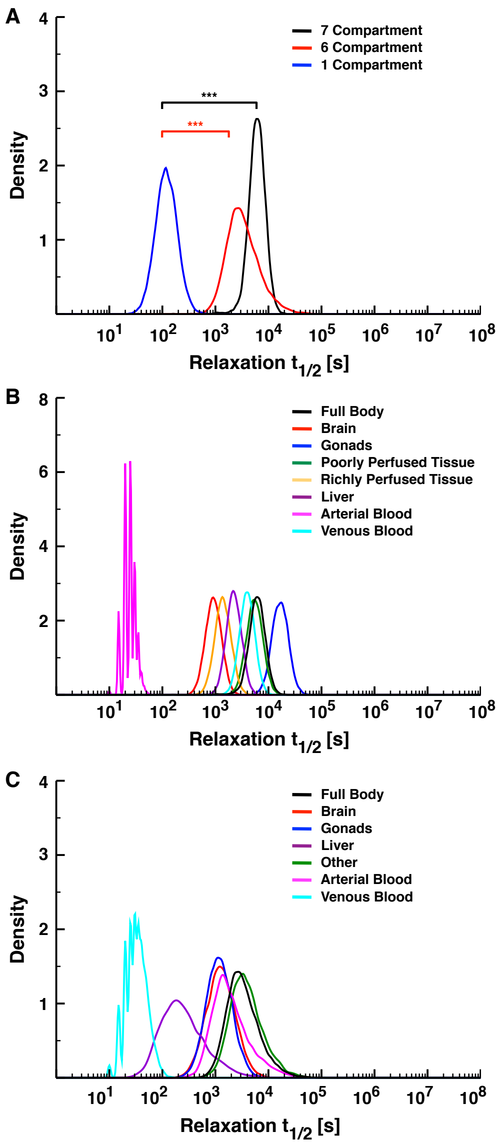

Figure S2: Relaxation dynamics of the PBTK models, exposure to 1,2-dichloroethane . (A) The kernel density plot of the relaxation half-lives of the whole-body chemical concentrations in the 7C (black), 6C (red), and 1C (blue) models. The sample populations of half-lives were taken from n=10^3 simulations varying the body parameters in a fashion similar to that described for Fig. 4 of the main text. The differences in the means from the 7C vs. 1C and 6C vs. 1C are statistically significant (p ¡ 0.01, Student's t-test). Note that the relaxation half-lives are plotted on a $\log_{10}$ scale. (B) The kernel density plot of the relaxation half-lives of the whole body (black) and tissue-specific chemical concentrations for the brain (red), gonads (blue), poorly perfused tissue (green), richly perfused tissue (orange), liver (purple), arterial blood (magenta), and venous blood (cyan). Note that the relaxation half-lives are plotted on a $\log_{10}$ scale. (C) The kernel density plot of the relaxation half-lives of the whole body (black) and tissue-specific chemical concentrations for the brain (red), gonads (blue), liver (purple), other tissues (green), arterial blood (magenta), and venous blood (cyan). Note that the relaxation half-lives are plotted on a $\log_{10}$ scale.

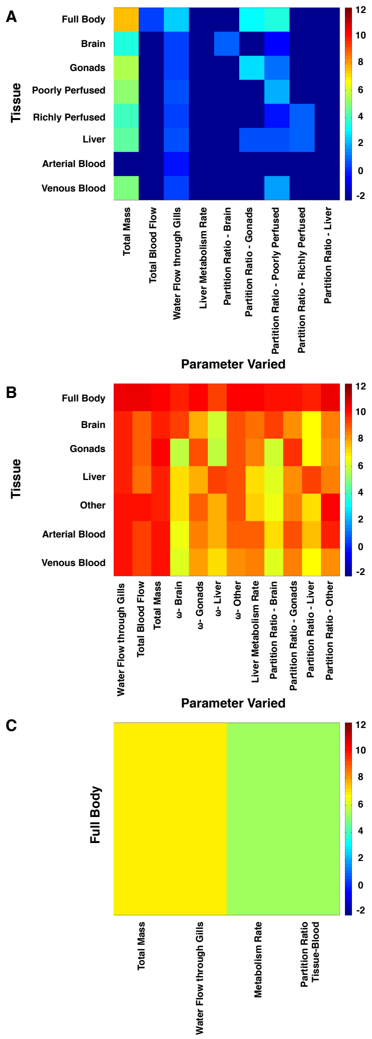

Figure S3: Relaxation dynamics sensitivity analyses. Each model was run 1000 times varying each of the indicated body parameters using a $\log_2$ normal distribution with an average equal to the literature-derived value similar to the sensitivity analyses presented in section 5. From these 1000 simulations we calculated the variance in the relaxation half-lives for the whole body and each of the individual tissue compartments. These variances are presented as a set of heat maps with the colorbar based on the $\log_{10}$ of the variance. The hotter the color, the more sensitive the relaxation of that compartment/whole body concentration is to changes in the magnitude of the parameter. The results for the 7C model are presented in *A*, 6C model in *B*, and 1C model in *C*.

2 Generalized Conceptual Model for an Organ with Metabolism

Before designing the physiologically-based toxicokinetic models, we must first examine the conceptual basis for such models. On an organismal scale, such models typically include different tissues and organs as a system of connected, well-mixed tanks (Fig. S4) [Peters, 2012]. The chemical is ingested and enters the organism's arterial blood. The chemical in the arterial blood can then be distributed to each of the organs. Some of the chemical can also leave the organs in venous blood, which can then potentially exit the body or be recycled into the arterial blood. Alternatively, in tissues and organs such as the liver and kidneys the chemical can either be transformed or metabolized, removing it from the system.

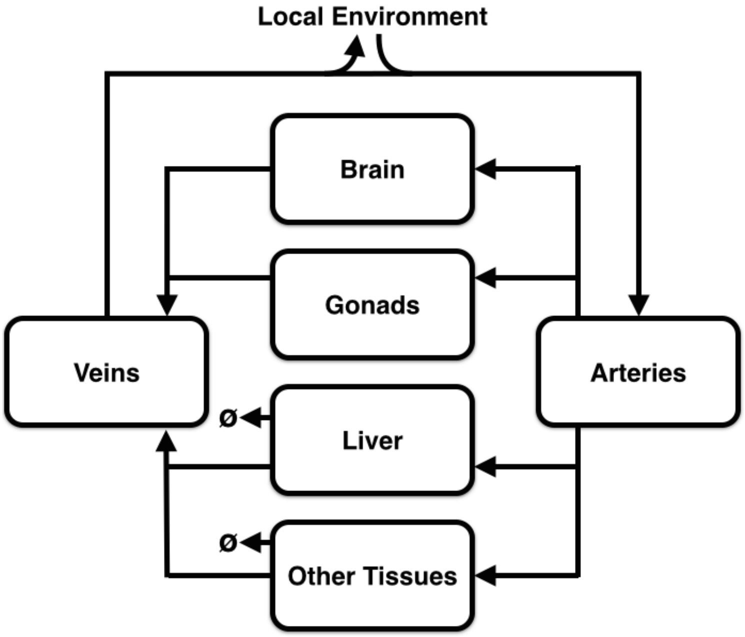

Figure S4: An example diagram depicting a well-mixed tank toxicokinetic model for an organism.

However, the organism-level depiction does not take into account the biological mechanics of transferring the chemical from the arterial blood into the organ or tissue and back into the venous blood or removed through biotransformation or metabolism. When a chemical in the arterial blood reaches a tissue, it must first filter down into the capillaries, which have thin walls to make transfer out of the blood easier (See Fig. S5). The chemical can then transfer from within the capillaries into the interstitial fluid and back. Chemical in the interstitial fluid can then enter the cell membranes and bulk cellular material and pass to and from the tissue cell's cytoplasm. It is within the cytoplasm in which the chemical may be metabolized, if that possibility exists for the given tissue.

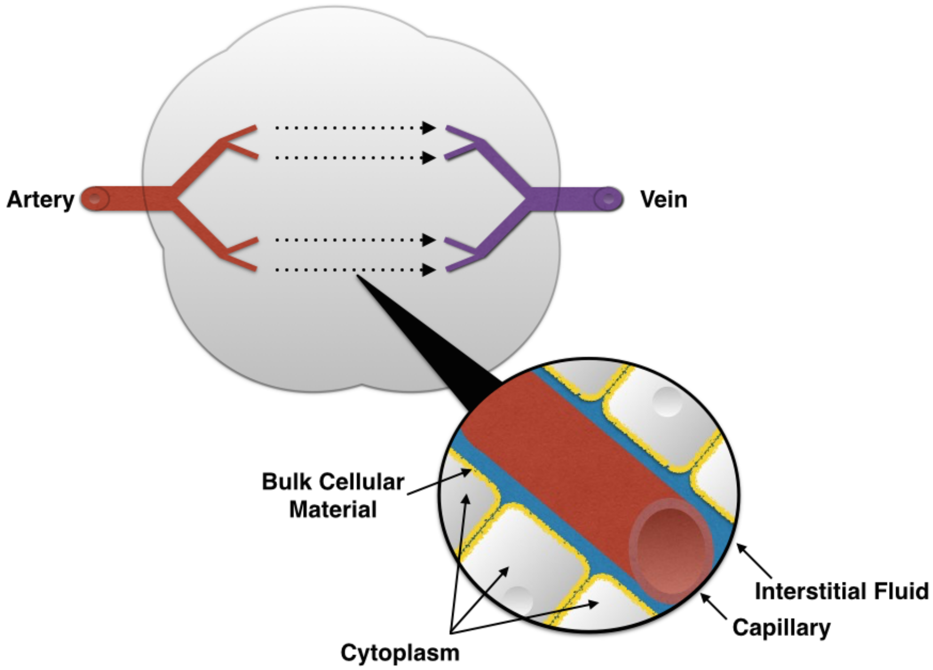

Figure S5: Cartoon depicting the relationship between the cardiovascular system (red and purple tubes and dotted arrows) and a generic organ (grey mass).

We can simplify the biological cartoon of Fig. S5 into a diagram (Fig. S6). Within this depiction the chemical localized in the subcompartments shaded grey (the capillary blood, interstitial fluid, bulk cellular material and cytoplasm) are considered to contribute to the total chemical concentration of the organ.

However, one thing to keep in mind is this model is supposed to be a single tank containing both bound (C_B) and free (C_{cap}, C_I and C_{cy}) chemical. The subcompartments as drawn in Fig. S6 represent different "states" of the chemical rather than actual, measurable volumes. As such, the change in the total amount of chemical in the organ can be described as:

$$V_T \frac{dC_T}{dt} = V_T \frac{dC_{cy}}{dt} + V_T \frac{dC_B}{dt} + V_T \frac{dC_I}{dt} + V_T \frac{dC_{cap}}{dt} \tag{S1}$$

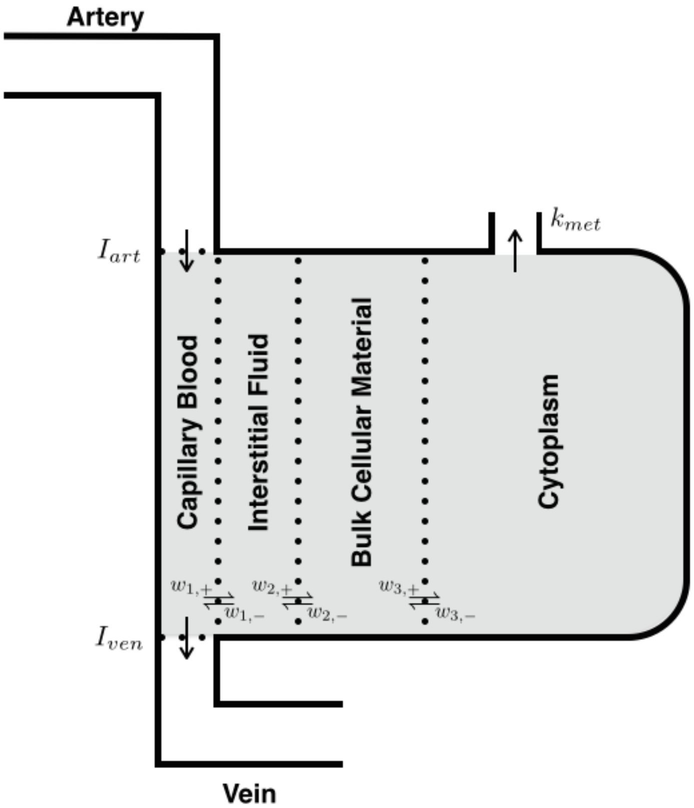

Figure S6: Cartoon depicting the relationship between the cardiovascular system (red and purple tubes and dotted arrows) and a generic organ (grey mass).

where V_T is the volume of the organ and C_T is the concentration of the chemical in the organ, and the remaining V and C terms represent the volumes and chemical concentrations in the cytoplasm (cy), bulk cellular material (B), interstitial fluid (I), and capillaries (cap). The contributing terms in Eq. S1 are defined as:

$$V_T \frac{dC_{cy}}{dt} = FC_{art} - FC_{cap} + C_I w_{1,-} - C_{cap} w_{1,+}$$
$$V_T \frac{dC_I}{dt} = C_{cap} w_{1,+} - C_I w_{1,-} + C_B w_{2,-} - C_{cap} w_{2,+}$$
$$V_T \frac{dC_B}{dt} = C_I w_{2,+} - C_B w_{2,-} + C_{cy} w_{3,-} - C_B w_{3,+}$$
$$V_T \frac{dC_{cy}}{dt} = C_B w_{3,+} - C_{cy} w_{3,-} - C_{cy} k_{met}$$

where F is rate of blood flow through the organ and C_{art} is the arterial concentration of the

chemical. These definitions can then be substituted into Eq. S1:

$$V_T \frac{dC_T}{dt} = FC_{art} - FC_{cap} - C_{cy}k_{met} \tag{S2}$$

If we assume chemicals in the interstitial fluid and cytoplasm are "free", then the concentrations of the chemical in the capillaries, interstitial fluid, and cytoplasm are equal at steady state [Peters, 2012]. We can then replace the variables C_{cap} and C_{cy} in Eq. S2 with C_{free}, where the new variable denotes the concentration of free chemical in the total organ, ignoring the distinction between subcompartments. This leaves us with the expression:

$$V_T \frac{dC_T}{dt} = FC_{art} - FC_{free} - C_{free}k_{met} \tag{S3}$$

For a final step, we want to relate the concentration of free chemical to the total concentration in the organ. This is done by the organ's tissue-blood ratio, P_T. P_T is the ratio of the total concentration of the chemical in the organ to the concentration of the chemical in the venous blood exiting the organ, and depends upon the water/octanol ratio of the chemical and the lipid and water content of the tissue. At steady state the concentration of the chemical in the exiting venous blood can be approximated by using the concentration of the chemical in the capillaries, or the free chemical concentration in the organ, C_{free}. As such, the tissue-blood ratio can be defined as:

$$P_T = \frac{C_T}{C_{free}}$$

which, in turn, can be substituted into Eq. S3:

$$V_T \frac{dC_T}{dt} = FC_{art} - \frac{F}{P_T}C_T - \frac{1}{P_T}C_T k_{met} \tag{S4}$$

From this we can obtain the ODE for any organ compartment that includes some form of metabolism or biotransformation. For organs without any means of chemical elimination besides returning it into the venous blood, the final term can be excluded.

3 Toxicokinetic model equations

3.1 7-Compartment PBTK model equations

The system of ODEs and parameters for a fish whose physiology is represented by 7 distinct, connected compartments. Its design is from the physiologically-based toxicokinetic model of Zebrafish (*Danio rerio*), based on the model and parameters given in Péry, *et al.* [Péry et al., 2013]. The change in the concentration of a chemical in organ i = brain, gonads, poorly perfused tissues, and richly perfused tissues via blood flows is described by:

$$\frac{dC_i}{dt} = \frac{F_i}{V_i}\left(C_{art} - \frac{C_i}{P_i}\right) \tag{S5}$$

where C_i is the chemical's concentration in organ i, C_{art} is the chemical's concentration in arterial blood, F_i is the fraction of blood flow in organ i, P_i is the partition coefficient between organ i and blood, and V_i is the volume of organ i. The change in concentration of a chemical in the liver, accounting for fish blood circulation and possible metabolism, is described by:

$$\frac{dC_{liv}}{dt} = \frac{F_{liv}}{V_{liv}}\left(C_{art} - \frac{C_{liv}}{P_{liv}}\right) + \frac{F_{gon}}{V_{liv}}\left(\frac{C_{gon}}{P_{gon}} - \frac{C_{liv}}{P_{liv}}\right) + \frac{F_{rpt}}{V_{liv}}\left(\frac{C_{rpt}}{P_{rpt}} - \frac{C_{liv}}{P_{liv}}\right) - \frac{k_{met}}{V_{liv}}\left(\frac{C_{liv}}{P_{liv}}\right) \quad \text{(S6)}$$

where the first term describes the change in concentration due to flow between liver and blood, and the second and third terms describes changes in concentration due to flows between liver and either the gonads or richly perfused tissues. The fourth term describes the removal of chemical from the system due to metabolism. The change in the chemical's concentration in arterial blood is described by:

$$\frac{dC_{art}}{dt} = \alpha\frac{F_{gill}}{V_{art}}C_{H_2O} - \left(\frac{F_{liv} + F_{gon} + F_{rpt} + F_{ppt} + F_{brn}}{V_{art}}\right)(C_{art} - C_{ven}) \quad \text{(S7)}$$

where the first term describes the change in concentration due to flow between blood in the gills and the surrounding water. The next term describes changes in chemical concentration in arterial blood due to flow into or from different organs. α is a the assimilation efficiency of the chemical and is defined by:

$$\alpha = \begin{cases} 0.577\log(K_{ow}) - 0.281 & \log(K_{ow}) < 4.7 \\ \log(0.8) & 4.7 \leq \log(K_{ow}) \leq 6.75 \\ -0.731\log(K_{ow}) + 4.84 & \log(K_{ow}) > 6.75 \end{cases}$$

Finally, the change in the chemicals' concentration in venous blood is described by:

$$\frac{dC_{ven}}{dt} = -\alpha\frac{F_{gill}}{V_{ven}P_{bw}}C_{ven} - \frac{F_{liv}}{V_{ven}}\left(C_{ven} - \frac{C_{liv}}{P_{liv}}\right) - \frac{F_{ggon}}{V_{ven}}\left(C_{ven} - \frac{C_{liv}}{P_{liv}}\right) \quad \text{(S8)}$$

$$- \frac{F_{rp}}{V_{ven}}\left(C_{ven} - \frac{C_{liv}}{P_{liv}}\right) - \frac{F_{ppt}}{V_{ven}}\left(C_{ven} - \frac{C_{ppt}}{P_{ppt}}\right) - \frac{F_{brn}}{V_{ven}}\left(C_{ven} - \frac{C_{brn}}{P_{brn}}\right) \quad \text{(S9)}$$

where the first term describes the change in concentration due to flow between venous blood and the gills and the subsequent terms describe changes due to flow from different organs. F_{bw} is the blood:water partition coefficient. F_{gill} is the flow rater of water through the gills.

The physiologically-based pharmacokinetic (PBPK) model includes 28 parameters. The body-specific parameters include:

Parameter	Description	Value	Unit
F_{brn}	Blood flow in brain	0.010175	μL/s
F_{gon}	Blood flow in gonads	0.01221	μL/s
F_{ppt}	Blood flow in poorly perfused tissues	0.12469	μL/s
F_{rpt}	Blood flow in richly perfused tissues	0.034225	μL/s
F_{liv}	Blood flow in liver	0.0037	μL/s
V_{art}	Volume of arterial blood	5.716	μL
V_{ven}	Volume of venous blood	11.431	μL
V_{brn}	Volume of brain	7.502	μL
V_{gon}	Volume of gonads	182.189	μL
V_{ppt}	Volume of poorly perfused tissues	788.771	μL
V_{rpt}	Volume of richly perfused tissues	53.585	μL
V_{liv}	Volume of liver	22.506	μL
F_{gill}	Volumetric flow rate of water through the gills	9.167	μL/s

The values for the blood flow in the different organs are calculated as:

$$F_{brn} \equiv f_{brn} f_{tot}$$
$$F_{gon} \equiv f_{gon} f_{tot}$$
$$F_{ppt} \equiv f_{tot} - F_{brn} - F_{gon} - F_{rpt} - F_{liv}$$
$$F_{rpt} \equiv f_{rpt} f_{tot}$$
$$F_{liv} \equiv f_{liv} f_{tot}$$

Where f_{tot} is the total blood flow rate throughout the body and f_i are the scaled blood flow rates for each tissue compartment. The values for the volume of the compartments are calculated as:

$$V_{art} \equiv 0.33 \frac{m_b m_{tot}}{\rho}$$
$$V_{ven} \equiv 0.67 \frac{m_b m_{tot}}{\rho}$$
$$V_{brn} \equiv \frac{m_{brn} m_{tot}}{\rho}$$
$$V_{gon} \equiv \frac{m_{gon} m_{tot}}{\rho}$$
$$V_{ppt} \equiv \frac{(1 - m_b - m_{brn} - mgon - m_{liv} - m_{rpt}) m_{tot}}{\rho}$$
$$V_{rpt} \equiv \frac{m_{rpt} m_{tot}}{\rho}$$
$$V_{liv} \equiv \frac{m_{liv} m_{tot}}{\rho}$$

where the value of the average density of the fish's body, ρ, is assumed to be 1 mg/μL. Finally, the value for F_{gill} is a simple scaling of f_{wat} to appropriate units:

$$F_{gill} = f_{wat} \times 10^3$$

Parameters given by Péry, *et al.* [Péry et al., 2013] include:

Parameter	Description	Value - male	Value - female	Unit
f_{car}	Cardiac output - (0.5g/26°C)	11.1	11.1	μL/min
f_{brn}	Scaled blood flow - brain	0.055	-	-
f_{gon}	Scaled blood flow - gonads	0.066	-	-
f_{rpt}	Scaled blood flow - richly perfused tissues	0.185	-	-
f_{liv}	Scaled blood flow - liver	0.02	-	-
m_{tot}	Total mass	617.6	1071.7	mg
m_b	Percentage of total mass - blood	1.6	1.6	-
m_{brn}	Percentage of total mass - brain	1.5	0.7	-
m_{gon}	Percentage of total mass - gonads	1.8	8 or **17**	-
m_{rpt}	Percentage of total mass - richly perfused tissues	5	5	-
m_{liv}	Percentage of total mass - liver	0.8	2.1	-
f_{wat}	Ventilation rate - (0.4g/27°C)	0.55	0.55	mL/min
W_{liv}	Water content - liver	0.65	0.69	-
W_{brn}	Water content - brain	0.75	0.76	-
W_{gon}	Water content - gonads	0.52	0.65	-
W_{tot}	Water content - total	0.68	0.65	-
L_{liv}	Lipid content - liver	0.105	0.059	-
L_{brn}	Lipid content - brain	0.073	0.076	-
L_{gon}	Lipid content - gonads	0.22	0.076	-
L_{oth}	Lipid content - rest of the body	0.046	0.046	-

We assume that the scaled blood flow in males and females are the same. The chemical-specific and simulation parameters include:

Parameter	Description	Units
C_{art}	Chemical concentration in arterial blood	μmol/μL
C_{ven}	Chemical concentration in venous blood	μmol/μL
C_{brn}	Chemical concentration in brain	μmol/μL
C_{gon}	Chemical concentration in gonads	μmol/μL
C_{ppt}	Chemical concentration in poorly perfused tissues	μmol/μL
C_{rpt}	Chemical concentration in richly perfused tissues	μmol/μL
C_{lib}	Chemical concentration in liver	μmol/μL
C_{H_2O}	Chemical concentration in water	μmol/μL
P_{brn}	Partition coefficient between brain and blood	-
P_{gon}	Partition coefficient between gonads and blood	-
P_{ppt}	Partition coefficient between poorly perfused tissues and blood	-
P_{rpt}	Partition coefficient between richly perfused tissues and blood	-
P_{liv}	Partition coefficient between liver and blood	-
k_{met}	Liver metabolism rate	s^{-1}
α	Assimilation efficiency	-
P_{bw}	Blood:water partition ratio	-

Additional values given by equations:

Description	Value
P_{bw}	$\log(P_{bw}) = 0.78\log(K_{ow}) - 0.82$
P_i	$\log(P_i$ - $W_i) = 0.74 \times \log(K_{ow}) + 1.00 \times \log(L_i) + 0.72$

We used two specific chemicals in this study: diazinon and 1,2-dichloroethane. Diazinon has a $\log_{10}$ octanol-water partition ratio of 3.81 and a liver clearance rate of 0.25 hr^{-1}. 1,2-Dichloroethane has a $\log_{10}$ octanol-water partition ratio of 1.48 and a clearance rate of 9 $\text{hr}^{-1}\text{kg}^{-1}$ [Hansch et al., 1995, D'Souza et al., 1987].

For Figure 3 of the main text the same parameters were randomly varied by selecting values from a log-normal distribution about the literature values. The exposure concentration was once again set to 10 μM and body concentrations initialized to 0. For Figure 5 of the main text we used randomly varied parameters using the same scheme as for Figure 3. Body concentrations were initialized to 0. Exposure concentration was initially set to 10 μM until the body concentrations reached steady state. The exposure concentration was then set to 10.1 μM and the simulations allowed to run until the body concentrations reached the new steady state. The exposure concentration was then reset to 10 μM until body concentrations reached their original steady state.

3.2 6-Compartment PBTK model equations

This model describes the concentrations of a chemical throughout the body of a fish divided into 6 distinct compartments in a large water bath. It is based off of the fathead minnow model developed by Li, *et al.* [Li et al., 2011]. The change in the concentration of the chemical in the brain is described by:

$$\frac{dC_{brn}}{dt} = \frac{F_{brn}}{V_{brn}}\left(C_{art,F} - \frac{C_{brn}}{P_{brn}}\right). \tag{S10}$$

The change in the concentration of the chemical in the gonads is similarly:

$$\frac{dC_{gon}}{dt} = \frac{F_{gon}}{V_{gon}}\left(C_{art,F} - \frac{C_{gon}}{P_{gon}}\right). \tag{S11}$$

The change in the concentration of the chemical in the liver is similar to Eqs. S10 and S12, but includes a term to account for metabolism of the chemical in the liver:

$$\frac{dC_{liv}}{dt} = \frac{F_{liv}}{V_{liv}}\left(C_{art,F} - \frac{C_{liv}}{P_{liv}}\right) - k_{liv}\frac{C_{liv}}{P_{liv}V_{liv}}. \tag{S12}$$

Finally, we have an ODE describing the change in the concentration of the chemical in the rest of the body, including potential excretion of the chemical:

$$\frac{dC_{oth}}{dt} = \frac{F_{oth}}{V_{oth}}\left(C_{art,F} - \frac{C_{oth}}{P_{oth}}\right) - k_{oth}\frac{C_{oth}}{P_{oth}V_{oth}}. \tag{S13}$$

The ODE describing the change in the concentration of the chemical in the arterial blood is:

$$\frac{dC_{art,F}}{dt} = \frac{F_{gill}}{V_{art}}C_{H_2O} + \frac{F_{car}}{V_{art}}C_{ven,F} - \frac{(F_{brn} + F_{gon} + F_{liv} + F_{oth})}{V_{art}}C_{art,F}. \tag{S14}$$

Additionally, we can define an ODE describing the change in the concentration of the chemical in the venous blood as:

$$\begin{aligned}\frac{dC_{ven,F}}{dt} = {} & \frac{F_{brn}}{V_{ven}P_{brn}}C_{brn} + \frac{F_{gon}}{V_{ven}P_{gon}}C_{gon} + \frac{F_{liv}}{V_{ven}P_{liv}}C_{liv} + \frac{F_{oth}}{V_{ven}P_{oth}}C_{oth} \\ & - \frac{F_{car}}{V_{ven}}C_{ven,F} - \frac{F_{gill}}{V_{ven}}C_{ven,F}.\end{aligned} \tag{S15}$$

Many of the parameters of these ODEs are calculated from input parameters and are defined as:

$$
\begin{aligned}
F_{gill} &\equiv F_{gill,max} m^{3/4} \\
F_{car} &\equiv F_{car,max} m^{3/4} \\
F_{brn} &\equiv \frac{\omega_{brn} f_{brn,BW}}{\sum_{tissues} \omega_i f_{i,BW}} F_{car} \\
F_{gon} &\equiv \frac{\omega_{gon} f_{gon,BW}}{\sum_{tissues} \omega_i f_{i,BW}} F_{car} \\
F_{liv} &\equiv \frac{\omega_{liv} f_{liv,BW}}{\sum_{tissues} \omega_i f_{i,BW}} F_{car} \\
F_{oth} &\equiv \left(1 - \frac{\omega_{brn} f_{brn,BW} + \omega_{gon} f_{gon,BW} + \omega_{liv} f_{liv,BW}}{\sum_{tissues} \omega_i f_{i,BW}}\right) F_{car} \\
\sum_{tissues} \omega_i f_{i,BW} &\equiv \omega_{brn} f_{brn,BW} + \omega_{gon} f_{gon,BW} + \omega_{liv} f_{liv,BW} + \omega_{oth} f_{oth,BW}
\end{aligned}
$$

$$
\begin{aligned}
f_{art,BW} &\equiv 0.5 * f_{ven,BW} \\
f_{oth,BW} &\equiv 1 - f_{brn,BW} - f_{gon,BW} - f_{liv,BW} - f_{gill,BW} - f_{art,BW} - f_{ven,BW} \\
V_{brn} &\equiv \frac{f_{brn,BW} m}{\rho} \\
V_{gon} &\equiv \frac{f_{gon,BW} m}{\rho} \\
V_{liv} &\equiv \frac{f_{liv,BW} m}{\rho} \\
V_{art} &\equiv \frac{f_{art,BW} m}{\rho} \\
V_{ven} &\equiv \frac{f_{ven,BW} m}{\rho} \\
V_{gill} &\equiv \frac{f_{gill,BW} m}{\rho} \\
V_{oth} &\equiv \frac{f_{oth,BW} m}{\rho}
\end{aligned}
$$

The physiological parameters for this model were combined from Péry, *et al.* and Li, *et al.* [Péry et al., 2013, Li et al., 2011] and include:

Parameter	Description	Value	Units
$F_{gill,max}$	Maximum water flow through gills	7.9436 x 10^{-2}	μL/s/mg$^{3/4}$
$F_{car,max}$	Maximum cardiac flow	9.8768 x 10^{-4}	μL/s/mg$^{3/4}$
m	Median value for female FHM mass	1071.7	mg
ρ	Average density of female FHM	1	mg/uL
$f_{brn,BW}$	Ratio of brain to body weight	0.0118	-
$f_{gon,BW}$	Ratio of gonad to body weight	0.11	-
$f_{liv,BW}$	Ratio of liver to body weight	0.0295	-
$f_{ven,BW}$	Ratio of venous blood to body weight	0.0167	-
$f_{gill,BW}$	Ratio of gill to body weight	0.0259	-
ω_{brn}	Tissue dependent weighting factor	0.036	-
ω_{gon}	Tissue dependent weighting factor	0.036	-
ω_{liv}	Tissue dependent weighting factor	0.024	-
ω_{oth}	Tissue dependent weighting factor	0.007	-

The chemical-specific parameters for this model include:

Parameter	Description	Units
P_{brn}	Ratio of brain to blood chemical concentration	-
P_{gon}	Ratio of gonad to blood chemical concentration	-
P_{liv}	Ratio of liver to blood chemical concentration	-
P_{gill}	Ratio of concentration of gill water to blood	-
P_{oth}	Ratio of other tissues to blood chemical concentration	-
k_{liv}	Rate of chemical metabolism (biotransformation/conjugation) by liver	s^{-1}
k_{oth}	Rate of chemical elimination by "other tissues"	s^{-1}

For Figure 3 of the main text the same parameters were randomly varied by selecting values from a log-normal distribution about the literature values. The exposure concentration was once again set to 10 μM and body concentrations initialized to 0. For Figure 5 of the main text we used randomly varied parameters using the same scheme as for Figure 3. Body concentrations were initialized to 0. Exposure concentration was initially set to 10 μM until the body concentrations reached steady state. The exposure concentration was then set to 10.1 μM and the simulations allowed to run until the body concentrations reached the new steady state. The exposure concentration was then reset to 10 μM until body concentrations reached their original steady state.

3.3 1-Compartment PBTK model equations

The third system we developed is the 1-Compartment PBTK model. In this model the fish is treated as a single compartment with gills. The change in chemical concentration in the body with respect to time is:

$$V_t \frac{dC_t}{dt} = -\frac{F_{gill}}{P_{bw}} C_t + F_{gill} C_{H_2O} - \frac{C_t}{P_t} k_{met}$$

V_t is the total volume of the fish, F_{gill} is the flow rate of water through the gills, P_{bw} is the blood-water partition ratio for the chemical, P_t is the tissue-blood partition ratio for the chemical and k_{met} is the metabolism/excretion rate for the chemical. We calculated P_{bw} and P_t as we had done for the 7-Compartment model based on the $\log_{10} K_{ow}$ and lipid and water content of the body (See Section 3.1). The values for the parameters include:

Parameter	Value	Unit
F_{gill}	14.8789577	μL/s
m_t	1071.7	mg
LC_t	0.050847	–
WC_t	0.628169	–
k_{met}	2.679 x 10^{-6}	s^{-1}

In Figure 4A and 6A of the main text, we varied the parameters with a log-normal distribution about the parameters given above.

4 Comparison of the 7-Compartment PBTK to the Zebrafish PBTK model

In order to simplify the mathematics of the Zebrafish PBTK model presented by Péry, *et al.* [Péry et al., 2013] we made two assumptions: (i) that the metabolism of chemical in the liver follow first-order mass action kinetics rather than the Michaelis kinetics described in the model by Péry, *et al.*, and (ii) the environmental concentration remains constant throughout the simulation. We compared the two models to see if the differences in liver metabolism kinetics significantly altered the response of the model to chemical exposure. We kept the same parameters throughout the model for diazinon as described in Section 3.1. The parameters for the Michaelis kinetics of liver metabolism were obtained from Péry, *et al.* [Péry et al., 2013].

Visually there is no discernible difference between the responses of the two models (Fig. S7). In order to quantify the similarity, we took the difference of the $\log_{10}$ transform of the two time series ($\Delta C(t) = \log_{10} C^Z(t) - \log_{10} C^7(t)$, where $C^Z(t)$ is the compartment's chemical concentration at time t for the Zebrafish model and $C^7(t)$ is the chemical concentration at time t for the 7-Compartment model). Using R, we then obtained a kernel density estimate for the set of $\Delta C(t)$ over all time points [R Core Team, 2015]. We then found the values of $\Delta C(t)$ that bounded 95% of the area under the kernel density estimate curve so that both tails included 2.5% of the area under the curve. If $\Delta C(t) = 0$ fell between these two values, then there would be no statistical evidence to suggest that there is a difference between the two time series. If it fell outside of these values then there is a significant difference between the curves ($p \leq 0.05$). These results are shown in Fig. S8, with the region representing 95% of the area under the density curve shaded grey and the average difference over all time points shown as a black vertical line. We found no instances in which there was a evidence supporting a difference in the responses of the two models.

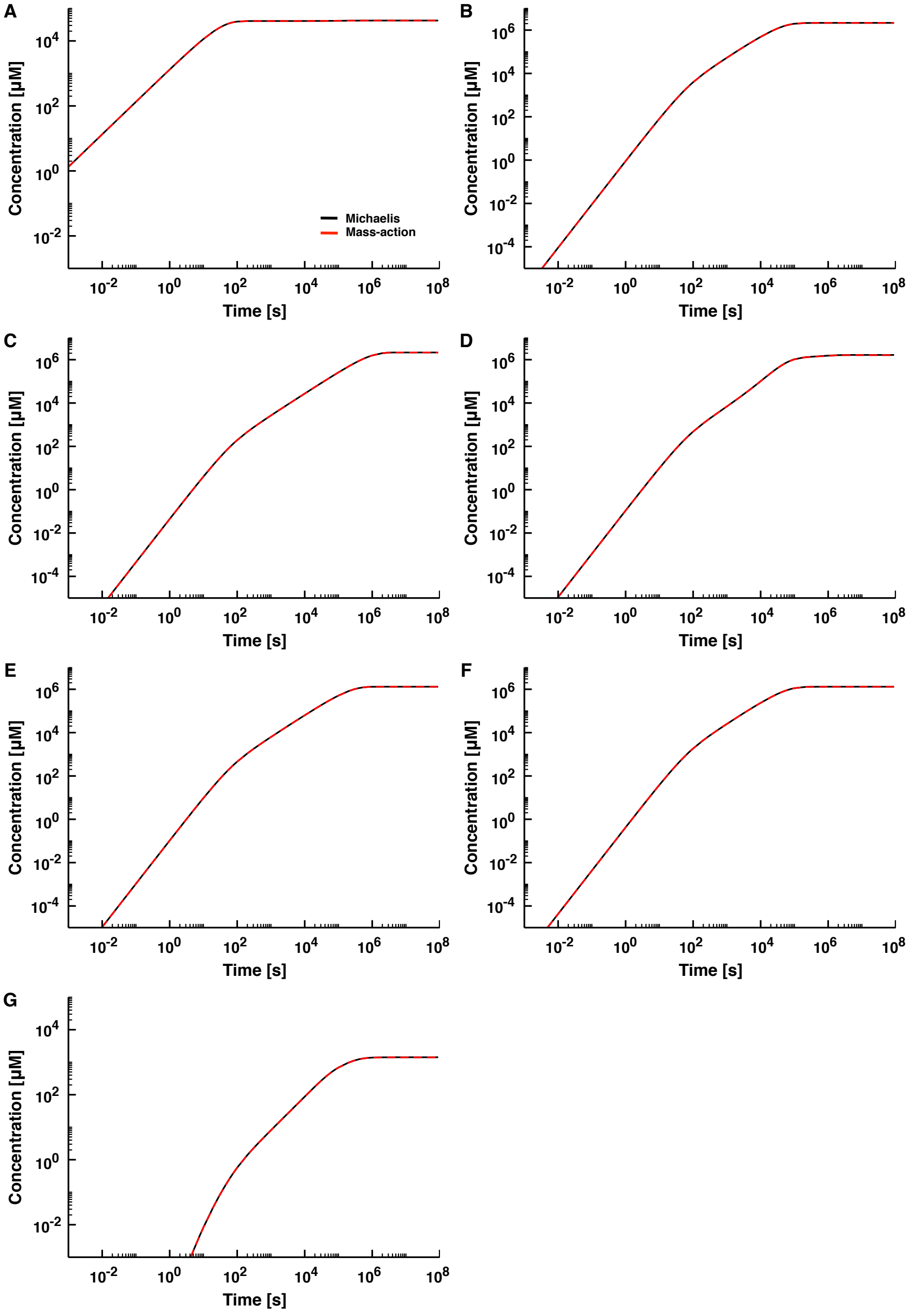

Figure S7: Accumulation of diazinon in different compartments as a function of time for the model described by Péry, *et al.* (with Michaelis kinetics governing liver metabolism, black) [Péry et al., 2013] and the 7-Compartment model (with mass action kinetics governing liver metabolism, red). Shown are accumulations in the arterial blood (A), brain (B), gonads (C), liver (D), poorly perfused tissue (E), richly perfused tissue (F), and venous blood (G).

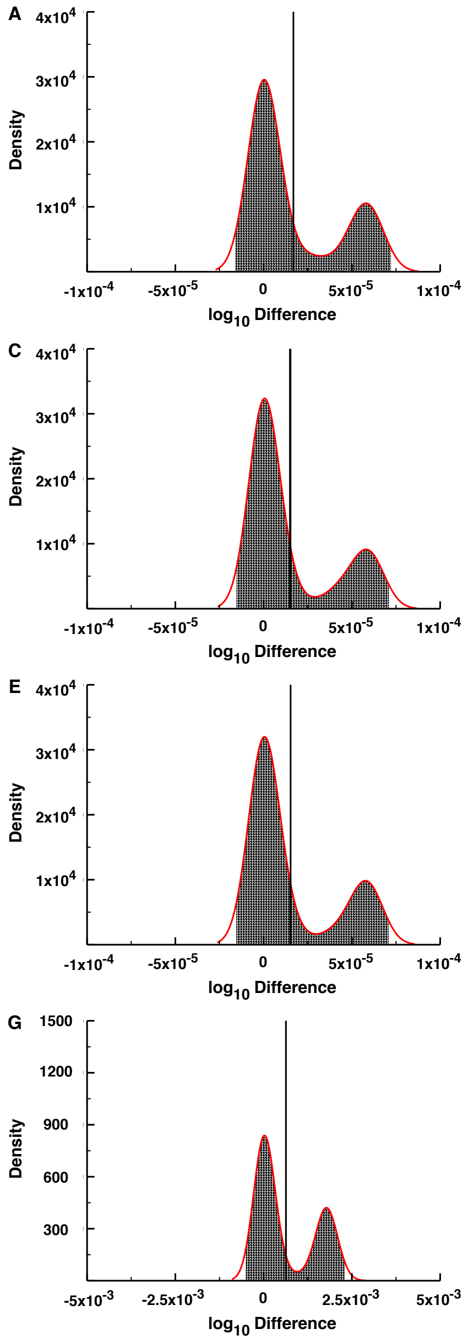

Figure S8: The distribution of differences of the $\log_{10}$ transformed time series in different compartments. Shown are accumulations in the arterial blood (A), brain (B), gonads (C), liver (D), poorly perfused tissue (E), richly perfused tissue (F), and venous blood (G). The grey shaded region indicates 95% of the area under the kernel density estimate curve and the black vertical line is the mean of the distribution.

5 Sensitivity of PBTK models to parameters

A sensitivity analysis was performed on the PBTK models evaluated at steady state by associating the variation in the model parameters to the variability in accumulated tissue concentrations (or PECs for the rTK models). We consider the individual parameter values to be log-normally distributed, and the mean for each distribution was set using literature sources while the standard deviation was fixed to 50% of the mean. The modeled organism was then exposed to a constant 10 μM diazinon (a model chemical with moderate octanol-water partition ratio) until the chemical concentrations in the organs reached steady state. We then recorded the ratio of the chemical concentration in each organ to the steady state chemical concentration in the respective organ in a simulation using literature-derived parameters (the distribution mean). The results are presented in Fig. S9 as a heat-map illustrating the variance in this ratio across all 10^6 simulations, wherein a higher variance indicates an increased sensitivity of the tissue concentration to the parameter.

The 7C model demonstrates minor sensitivities to blood-flow rate into the brain, gonads, poorly perfused tissue, and richly perfused tissue (Fig. S9A). The largest impact stems from the water-flow rate through gills and organ-specific partition ratio. Similarly, the 6C displayed some sensitivity to the water flow through the gills and the total blood flow rate (Fig. S9B). However, partition ratios for the brain, gonads, liver and other tissues, exhibited greater impact on their respective compartments. The 1C model demonstrated no distinguishable sensitivities to any of the 5 tested parameters (Fig. S9C).

We can better understand these results by examining the analytical form of the steady state concentration, which we provide for the 6C and 7C models in terms of the environmental chemical concentration (Sec. 3.1, 3.2, 3.3). We find that these expressions depend heavily upon the blood flow rates, the water flow rate through gills, and tissue-blood partition ratios. That the models are sensitive to partition ratios is intuitive, as their value increases with chemical hydrophobicity and tissue lipid content. If hydrophobicity is held constant, then an increase in the partition ratio suggests a greater tissue lipid content, allowing it to more readily sequester absorbed chemical from distribution to other tissues via the circulatory system.

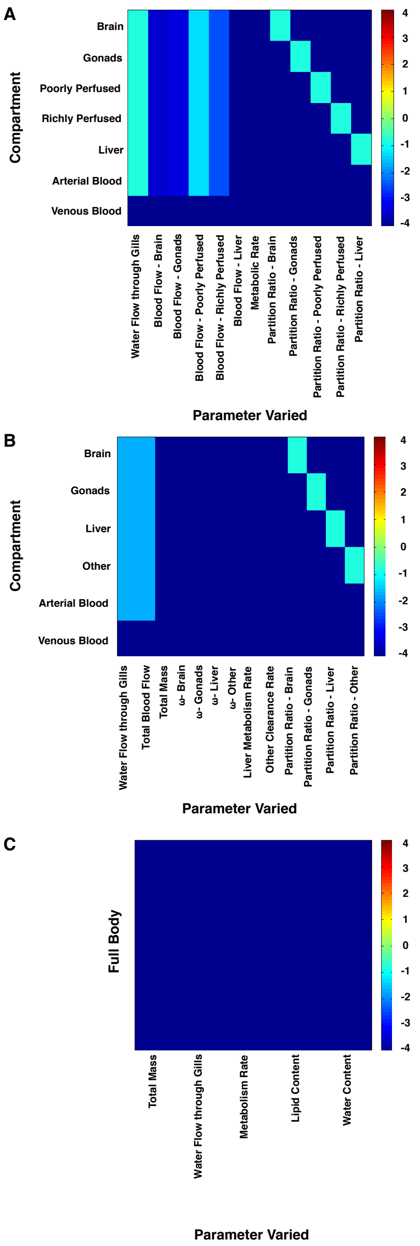

Figure S9: Sensitivity analyses of the PBTK models. For each model we independently varied a series of body-specific parameters using a log-normal distribution about the literature-derived value. Each parameter is varied randomly n=10^4 times, and the steady state chemical concentration in each compartment is compared against the steady state chemical concentrations found when using only the literature-derived values of the parameters. The heat maps pictured here represent the variance in the ratio of varied vs. literature-derived steady states over the 10^4 simulations for each compartment in response to changes in each parameter changed. The higher the variance, the more sensitive that compartment is to changes in that particular parameter. Note that the variances are shown here on a $\log_{10}$ scale. (A) The variances in steady states for the 7C model. (B) The variances in steady states for the 6C model. (C) The variances in steady states for the 1C model.

6 Reverse toxicokinetic model equations

6.1 7-compartment rTK model equations

We can place the system of ODEs for the 7-component PBTK model given in the previous section into a pseudo-steady state by setting each equation equal to zero:

$$
\begin{aligned}
\frac{dC_{brn}}{dt} &= \frac{F_{brn}}{V_{brn}}\left(C_{art} - \frac{C_{brn}}{P_{brn}}\right) = 0\\
\frac{dC_{gon}}{dt} &= \frac{F_{gon}}{V_{gon}}\left(C_{art} - \frac{C_{gon}}{P_{gon}}\right) = 0\\
\frac{dC_{ppt}}{dt} &= \frac{F_{ppt}}{V_{ppt}}\left(C_{art} - \frac{C_{ppt}}{P_{ppt}}\right) = 0\\
\frac{dC_{rpt}}{dt} &= \frac{F_{rpt}}{V_{rpt}}\left(C_{art} - \frac{C_{rpt}}{P_{rpt}}\right) = 0\\
\frac{dC_{liv}}{dt} &= \frac{F_{liv}}{V_{liv}}\left(C_{art} - \frac{C_{liv}}{P_{liv}}\right) + \frac{F_{gon}}{V_{liv}}\left(\frac{C_{gon}}{P_{gon}} - \frac{C_{liv}}{P_{liv}}\right) + \frac{F_{rpt}}{V_{liv}}\left(\frac{C_{rpt}}{P_{rpt}} - \frac{C_{liv}}{P_{liv}}\right) - \frac{k_{met}}{V_{liv}}\left(\frac{C_{liv}}{P_{liv}}\right) = 0\\
\frac{dC_{art}}{dt} &= \alpha\frac{F_{gill}}{V_{art}}C_{H_2O} - \left(\frac{F_{brn} + F_{gon} + F_{ppt} + F_{rpt} + F_{liv}}{V_{art}}\right)(C_{art} - C_{ven}) = 0\\
\frac{dC_{ven}}{dt} &= -\alpha\frac{F_{gill}}{V_{ven}P_{bw}}C_{ven} - \frac{F_{liv}}{V_{ven}}\left(C_{ven} - \frac{C_{liv}}{P_{liv}}\right) - \frac{F_{gon}}{V_{ven}}\left(C_{ven} - \frac{C_{liv}}{P_{liv}}\right) - \frac{F_{rpt}}{V_{ven}}\left(C_{ven} - \frac{C_{liv}}{P_{liv}}\right)\\
&\quad - \frac{F_{ppt}}{V_{ven}}\left(C_{ven} - \frac{C_{ppt}}{P_{ppt}}\right) - \frac{F_{brn}}{V_{ven}}\left(C_{ven} - \frac{C_{brn}}{P_{brn}}\right) = 0
\end{aligned}
$$

We can then solve each of these equations so that we can relate the concentrations within the blood and tissues to the concentration in the water. This can be shown best in matrix form:

$$\mathbf{AC} = \mathbf{W}, \tag{S16}$$

where

$$
\mathbf{A} = \begin{bmatrix}
-F_{brn}/P_{brn} & 0 & 0 & 0 & 0 & F_{brn} & 0\\
0 & -F_{gon}/P_{gon} & 0 & 0 & 0 & F_{gon} & 0\\
0 & 0 & -F_{ppt}/P_{ppt} & 0 & 0 & F_{ppt} & 0\\
0 & 0 & 0 & -F_{rpt}/P_{rpt} & 0 & F_{rpt} & 0\\
0 & F_{gon}/P_{gon} & 0 & F_{rpt}/P_{rpt} & p_{liv,liv} & F_{liv} & 0\\
0 & 0 & 0 & 0 & 0 & -F_{car} & F_{car}\\
F_{brn}/P_{brn} & 0 & F_{ppt}/P_{ppt} & 0 & p_{ven,liv} & 0 & -F_{car} - \alpha F_{gill}/P_{bw}
\end{bmatrix},
$$

$$\mathbf{C} = \begin{bmatrix} C_{brn} \\ C_{gon} \\ C_{ppt} \\ C_{rpt} \\ C_{liv} \\ C_{art} \\ C_{ven} \end{bmatrix},$$

and

$$\mathbf{W} = \begin{bmatrix} 0 \\ 0 \\ 0 \\ 0 \\ 0 \\ -\alpha F_{gill} \\ 0 \end{bmatrix}.$$

For simplicity, we have defined the following quantities:

$$p_{liv,liv} \equiv -F_{liv}/P_{liv} - F_{gon}/P_{liv} - F_{rpt}/P_{liv} - k_{met}/P_{liv}$$
$$p_{ven,liv} \equiv F_{liv}/P_{liv} + F_{gon}/P_{liv} + F_{rpt}/P_{liv}$$

We can then solve this system of equations to find the concentration of the chemical in the blood and tissues at steady state:

$$C_{brn} = \frac{\alpha F_{gill}(F_{gon} + F_{rpt} + F_{liv} + k_{met})(\alpha F_{gill} + P_{bw}F_{car})P_{brn}C_{H_2O}}{F_{car}(\alpha F_{gill}(F_{gon} + F_{rpt} + F_{liv} + k_{met}) + P_{bw}k_{met}(F_{gon} + F_{rpt} + F_{liv}))} \tag{S17}$$

$$C_{gon} = \frac{\alpha F_{gill}(F_{gon} + F_{rpt} + F_{liv} + k_{met})(\alpha F_{gill} + P_{bw}F_{car})P_{gon}C_{H_2O}}{F_{car}(\alpha F_{gill}(F_{gon} + F_{rpt} + F_{liv} + k_{met}) + P_{bw}k_{met}(F_{gon} + F_{rpt} + F_{liv}))} \tag{S18}$$

$$C_{ppt} = \frac{\alpha F_{gill}(F_{gon} + F_{rpt} + F_{liv} + k_{met})(\alpha F_{gill} + F_{bw}F_{car})P_{ppt}C_{H_2O}}{F_{car}(\alpha F_{gill}(F_{gon} + F_{rpt} + F_{liv} + k_{met}) + P_{bw}k_{met}(F_{gon} + F_{rpt} + F_{liv}))} \tag{S19}$$

$$C_{rpt} = \frac{\alpha F_{gill}(F_{gon} + F_{rpt} + F_{liv} + k_{met})(\alpha F_{gill} + P_{bw}F_{car})P_{rpt}C_{H_2O}}{F_{car}(\alpha F_{gill}(F_{gon} + F_{rpt} + F_{liv} + k_{met}) + P_{bw}k_{met}(F_{gon} + F_{rpt} + F_{liv}))} \tag{S20}$$

$$C_{liv} = \frac{\alpha F_{gill}(F_{gon} + F_{rpt} + F_{liv})(\alpha F_{gill} + P_{bw}F_{car})P_{liv}C_{H_2O}}{F_{car}(\alpha F_{gill}(F_{gon} + F_{rpt} + F_{liv} + k_{met}) + P_{bw}k_{met}(F_{gon} + F_{rpt} + F_{liv}))} \tag{S21}$$

$$C_{art} = \frac{\alpha F_{gill}(F_{gon} + F_{rpt} + F_{liv} + k_{met})(\alpha F_{gill} + P_{bw}F_{car})C_{H_2O}}{F_{car}(\alpha F_{gill}(F_{gon} + F_{rpt} + F_{liv} + k_{met}) + P_{bw}k_{met}(F_{gon} + F_{rpt} + F_{liv}))} \tag{S22}$$

$$C_{ven} = \frac{\alpha F_{gill}((F_{gon} + F_{rpt} + F_{liv})F_{car} + k_{met}(F_{brn} + F_{ppt}))F_{bw}C_{H_2O}}{F_{car}(\alpha F_{gill}(F_{gon} + F_{rpt} + F_{liv} + k_{met}) + P_{bw}k_{met}(F_{gon} + F_{rpt} + F_{liv}))} \tag{S23}$$

The concentrations C_i in these expressions are now understood as referring to steady-state concentrations. From these equations we can easily obtain an inverted model describing an estimated concentration of the chemical in the water based on the chemical concentration in any

particular tissues by solving each equation for C_{H_2O}:

$$C_{H_2O} = \frac{D_1 C_{brn}}{D_2 P_{brn}} \tag{S24}$$

$$C_{H_2O} = \frac{D_1 C_{gon}}{D_2 P_{gon}} \tag{S25}$$

$$C_{H_2O} = \frac{D_1 C_{ppt}}{D_2 P_{ppt}} \tag{S26}$$

$$C_{H_2O} = \frac{D_1 C_{rpt}}{D_2 P_{rpt}} \tag{S27}$$

$$C_{H_2O} = \frac{D_1 C_{liv}}{D_3 P_{liv}} \tag{S28}$$

$$C_{H_2O} = \frac{D_1 C_{art}}{D_2} \tag{S29}$$

$$C_{H_2O} = \frac{D_1 C_{ven}}{D_4} \tag{S30}$$

Where we have defined the following expressions for simplicity:

$$D_1 \equiv F_{car}(\alpha F_{gill}(F_{gon} + F_{rpt} + F_{liv} + k_{met}) + P_{bw} k_{met}(F_{gon} + F_{rpt} + F_{liv})$$

$$D_2 \equiv \alpha F_{gill}(F_{gon} + F_{rpt} + F_{liv} + k_{met})(\alpha F_{gill} + P_{bw} F_{car})$$

$$D_3 \equiv \alpha F_{gill}(F_{gon} + F_{rpt} + F_{liv})(\alpha F_{gill} + P_{bw} F_{car})$$

$$D_4 \equiv alpha F_{gill}((F_{gon} + F_{rpt} + F_{liv})F_{car} + k_{met}(F_{brn} + F_{ppt}))F_{bw}$$

We can also represent these expressions in the form:

$$C_{H_2O} = A_i C_i$$

where, for the expression based upon the chemical concentration in the brain:

$$A_{brn} = \frac{D_1}{D_2 P_{brn}}$$

We can then define an expression for the exposure concentration in terms of the total body concentration as:

$$C_{H_2O} = C_{tot} V_{tot} / \left(\sum_i V_i / A_i \right)$$

Where $i = brn, gon, ppt, rpt, liv, art, ven$.

For Figure 6 of the main text the model was also parameterized using literature derived values as listed in Section 3.1. The $\log_{10}$ K_{ow} and k_{met} values were then varied between -2 – 10 and 10^{-7} – 10^{-2}, respectively.

6.2 6-compartment rTK model equations

We can place the system of ODEs for the 6-compartment PBTK model given in the previous section into a pseudo-steady state by setting each equation equal to zero:

$$
\begin{aligned}
\frac{dC_{brn}}{dt} &= \frac{F_{brn}}{V_{brn}}\left(C_{art,F} - \frac{C_{brn}}{P_{brn}}\right) = 0 \\
\frac{dC_{gon}}{dt} &= \frac{F_{gon}}{V_{gon}}\left(C_{art,F} - \frac{C_{gon}}{P_{gon}}\right) = 0 \\
\frac{dC_{liv}}{dt} &= \frac{F_{liv}}{V_{liv}}\left(C_{art,F} - \frac{C_{liv}}{P_{liv}}\right) - k_{liv}\frac{C_{liv}}{P_{liv}} = 0 \\
\frac{dC_{oth}}{dt} &= \frac{F_{oth}}{V_{oth}}\left(C_{art,F} - \frac{C_{oth}}{P_{oth}}\right) - k_{oth}\frac{C_{oth}}{P_{oth}} = 0 \\
\frac{dC_{art,F}}{dt} &= \frac{F_{gill}}{V_{art}}C_{H_2O} + \frac{F_{car}}{V_{art}}C_{ven,F} - \frac{F_{car}}{V_{art}}C_{art,F} = 0 \\
\frac{dC_{ven,F}}{dt} &= \frac{F_{brn}}{V_{ven}P_{brn}}C_{brn} + \frac{F_{gon}}{V_{ven}P_{gon}}C_{gon} + \frac{F_{liv}}{V_{ven}P_{liv}}C_{liv} + \frac{F_{oth}}{V_{ven}P_{oth}}C_{oth} - \frac{F_{car} + F_{gill}}{V_{ven}}C_{ven,F} = 0
\end{aligned}
$$

Note that we simplified the expression for $\frac{dC_{art,F}}{dt}$ by using the definition $F_{car} \equiv F_{brn} + F_{gon} + F_{liv} + F_{oth}$ from the auxiliary equations for calculating parameters. We can then solve each of these equations so that we can relate the concentrations within the blood and tissues to the concentration in the water. This can be shown in matrix form:

$$\mathbf{AC} = \mathbf{W} \tag{S31}$$

Where

$$
\mathbf{A} = \begin{bmatrix}
-F_{brn}/P_{brn} & 0 & 0 & 0 & F_{brn} & 0 \\
0 & -F_{gon}/P_{gon} & 0 & 0 & F_{gon} & 0 \\
0 & 0 & -(F_{liv} + k_{liv})/P_{liv} & 0 & F_{liv} & 0 \\
0 & 0 & 0 & -(F_{oth} + k_{oth})/P_{oth} & F_{oth} & 0 \\
0 & 0 & 0 & 0 & -F_{car} & F_{car} \\
F_{brn}/P_{brn} & F_{gon}/P_{gon} & F_{liv}/P_{liv} & F_{oth}/P_{oth} & 0 & -(F_{car} + (F_{gill}/P_{bw}))
\end{bmatrix},
$$

$$
\mathbf{C} = \begin{bmatrix} C_{brn} \\ C_{gon} \\ C_{liv} \\ C_{oth} \\ C_{art,F} \\ C_{ven,F} \end{bmatrix},
$$

and

$$\mathbf{W} = \begin{bmatrix} 0 \\ 0 \\ 0 \\ 0 \\ -F_{gill} \\ 0 \end{bmatrix}.$$

We can then solve this system of equations to find the concentration of the chemical in the blood and tissues at steady state:

$$C_{brn} = \frac{F_{gill}(F_{liv}+k_{liv})(F_{oth}+k_{oth})(P_{bw}F_{car}+F_{gill})P_{brn}C_{H_2O}}{F_{car}(F_{gill}(F_{liv}+k_{liv})(F_{oth}+k_{oth})+P_{bw}(F_{liv}(F_{oth}(k_{liv}+k_{oth})+k_{liv}k_{oth})+F_{oth}k_{liv}k_{oth}))} \tag{S32}$$

$$C_{gon} = \frac{F_{gill}(F_{liv}+k_{liv})(F_{oth}+k_{oth})(P_{bw}F_{car}+F_{gill})P_{gon}C_{H_2O}}{F_{car}(F_{gill}(F_{liv}+k_{liv})(F_{oth}+k_{oth})+P_{bw}(F_{liv}(F_{oth}(k_{liv}+k_{oth})+k_{liv}k_{oth})+F_{oth}k_{liv}k_{oth}))} \tag{S33}$$

$$C_{liv} = \frac{F_{gill}F_{liv}(F_{oth}+k_{oth})(P_{bw}F_{car}+F_{gill})P_{liv}C_{H_2O}}{F_{car}(F_{gill}(F_{liv}+k_{liv})(F_{oth}+k_{oth})+P_{bw}(F_{liv}(F_{oth}(k_{liv}+k_{oth})+k_{liv}k_{oth})+F_{oth}k_{liv}k_{oth}))} \tag{S34}$$

$$C_{oth} = \frac{F_{gill}(F_{liv}+k_{liv})F_{oth}(P_{bw}F_{car}+F_{gill})P_{oth}C_{H_2O}}{F_{car}(F_{gill}(F_{liv}+k_{liv})(F_{oth}+k_{oth})+P_{bw}(F_{liv}(F_{oth}(k_{liv}+k_{oth})+k_{liv}k_{oth})+F_{oth}k_{liv}k_{oth}))} \tag{S35}$$

$$C_{art,F} = \frac{F_{gill}(F_{liv}+k_{liv})(F_{oth}+k_{oth})(P_{bw}F_{car}+F_{gill})C_{H_2O}}{F_{car}(F_{gill}(F_{liv}+k_{liv})(F_{oth}+k_{oth})+P_{bw}(F_{liv}(F_{oth}(k_{liv}+k_{oth})+k_{liv}k_{oth})+F_{oth}k_{liv}k_{oth}))} \tag{S36}$$

$$C_{ven,F} = \frac{F_{gill}P_{bw}(F_{liv}F_{oth}F_{car}+k_{oth}(F_{liv}(F_{brn}+F_{gon}+F_{liv})+k_{liv}(F_{brn}+F_{gon}))+F_{oth}k_{liv}(F_{brn}+F_{gon}+F_{oth}))C_{H_2O}}{F_{car}(F_{gill}(F_{liv}+k_{liv})(F_{oth}+k_{oth})+P_{bw}(F_{liv}(F_{oth}(k_{liv}+k_{oth})+k_{liv}k_{oth})+F_{oth}k_{liv}k_{oth}))} \tag{S37}$$

From these equations we can easily obtain an inverted model describing an estimated concentration of the chemical in the water based on the chemical concentration in any particular tissue by solving each equation for C_{H_2O}:

$$C_{H_2O} = \frac{D_5 C_{brn}}{D_6 P_{brn}} \tag{S38}$$

$$C_{H_2O} = \frac{D_5 C_{gon}}{D_6 P_{gon}} \tag{S39}$$

$$C_{H_2O} = \frac{D_5 C_{liv}}{D_7 P_{liv}} \tag{S40}$$

$$C_{H_2O} = \frac{D_5 C_{oth}}{D_8 P_{oth}} \tag{S41}$$

$$C_{H_2O} = \frac{D_5 C_{art,F}}{D_6} \tag{S42}$$

$$C_{H_2O} = \frac{D_9 C_{ven,F}}{D_{10}} \tag{S43}$$

We have defined the following terms for simplicity:

$$
\begin{aligned}
D_5 &\equiv F_{car}(F_{gill}(F_{liv}+k_{liv})(F_{oth}+k_{oth})+P_{bw}(F_{liv}(F_{oth}(k_{liv}+k_{oth})+k_{liv}k_{oth})+F_{oth}k_{liv}k_{oth}))\\
D_6 &\equiv F_{gill}(F_{liv}+k_{liv})(F_{oth}+k_{oth})(P_{bw}F_{car}+F_{gill})\\
D_7 &\equiv F_{gill}F_{liv}(F_{oth}+k_{oth})(P_{bw}F_{car}+F_{gill})\\
D_8 &\equiv F_{gill}F_{oth}(F_{liv}+k_{liv})(P_{bw}F_{car}+F_{gill})\\
D_9 &\equiv F_{car}(F_{gill}(F_{liv}+k_{liv})(F_{oth}+k_{oth})+P_{bw}(F_{liv}(F_{oth}(k_{liv}+k_{oth})+k_{liv}k_{oth})+F_{oth}k_{liv}k_{oth}))\\
D_{10} &\equiv F_{gill}P_{bw}(F_{liv}F_{oth}F_{car}+k_{oth}(F_{liv}(F_{brn}+F_{gon}+F_{liv})\\
&\quad +k_{liv}(F_{brn}+F_{gon}))+F_{oth}k_{liv}(F_{brn}+F_{gon}+F_{oth}))
\end{aligned}
$$

We can also represent these expressions in the form:

$$C_{H_2O} = A_i C_i$$

where, for the expression based upon the chemical concentration in the brain:

$$A_{brn} = \frac{D_5}{D_6 P_{brn}}$$

We can then define an expression for the exposure concentration in terms of the total body concentration as:

$$C_{H_2O} = C_{tot}V_{tot}/\left(\sum_i V_i/A_i\right)$$

Where $i = brn, gon, liv, oth, art, ven$.

For Figure 7 of the main text the model was also parameterized using literature derived values as listed in Section 3.2. The $\log_{10} K_{ow}$ and k_{liv}/k_{oth} values were then varied between -2 – 10 and $10^{-7} – 10^{-2}$, respectively.

6.3 1-Compartment rTK model equations

The third system we developed is the 1-Compartment PBTK model. In this model the fish is treated as a single compartment with gills. The change in chemical concentration in the body with respect to time is:

$$V_t \frac{dC_t}{dt} = -\frac{F_{gill}}{P_{bw}} C_t + F_{gill} C_{H_2O} - \frac{C_t}{P_t} k_{met}$$

We then derived an expression for the exposure concentration as a function of the body concentration:

$$C_{H_2O} = \frac{((P_{bw} * k_{met}) + (F_{gill} P_t)) C_t}{F_{gill} P_{bw} P_t}$$

For Figure 7 of the main text we used the reverse toxicokinetic model given above with the parameter values given above.

7 Influence of Architecture on Steady State Compartment Concentrations

There are differences in the kinetics and architecture of the different models. For example, only the 7-compartment model includes an assimilation efficiency (α) term in the flow of chemical to/from the gills (See Section 3.1). To investigate how architecture itself can influence the steady state of the compartments within the body, we developed a series of three basic PBTK models. The first model is similar to the 6-compartment model (See Section 3.2) in that it has separate compartments for the brain, liver, and other tissues. In this case, however, only the liver can metabolize or excrete chemical, and the gonads are included within the other tissues. The diagram for this model is:

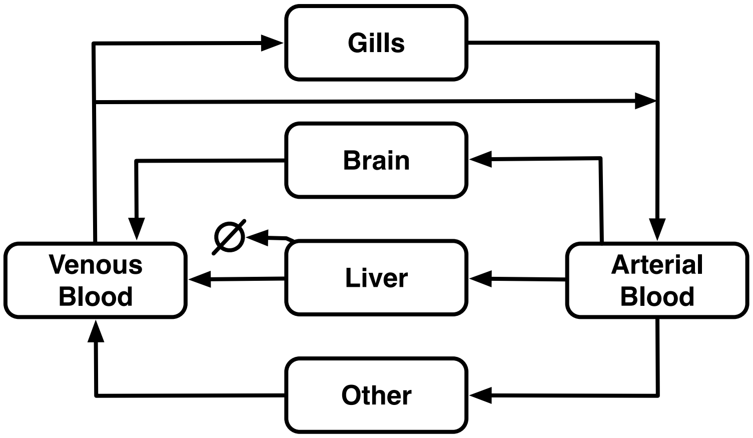

The system of ODEs describing the accumulation of chemical in the compartments are:

$$
\begin{aligned}
V_{brn}\frac{dC_{brn,1}}{dt} &= F_{brn}\left(C_{art,1} - \frac{C_{brn,1}}{P_{brn}}\right) \\
V_{liv}\frac{dC_{liv,1}}{dt} &= F_{liv}\left(C_{art,1} - \frac{C_{liv,1}}{P_{liv}}\right) - \frac{C_{liv,1}}{P_{liv}}k_{met} \\
V_{oth,1}\frac{dC_{oth,1}}{dt} &= F_{oth,1}\left(C_{art,1} - \frac{C_{oth,1}}{P_{oth,1}}\right) \\
V_{art}\frac{dC_{art,1}}{dt} &= \frac{F_{gill}}{P_{bw}}C_{H_2O} - F_{car}(C_{art,1} - C_{ven,1}) \\
V_{ven}\frac{dC_{ven,1}}{dt} &= -\frac{F_{gill}}{P_{bw}}C_{ven,1} - F_{car}C_{ven,1} + F_{brn}\frac{C_{brn,1}}{P_{brn}} + F_{liv}\frac{C_{liv,1}}{P_{liv}} + F_{oth,1}\frac{C_{oth,1}}{P_{oth,1}}
\end{aligned}
$$

where C_{H_2O} is the exposure concentration in the water, P_{bw} is the blood-water partition ratio, and C_i, V_i, and P_i are the chemical concentrations, volume, and partition ratios of the various compartments $i = brn, liv, oth, art, ven$. F_{brn}, F_{liv}, and F_{oth} represent the blood flow rate through the brain, liver and other tissues, respectively. F_{car} is the total cardiac flow through the body and is the sum: $F_{car} = F_{brn} + F_{liv} + F_{oth,1}$. F_{gill} is the flow rate of water through the gills. Finally, k_{met} is the metabolism rate of chemical in the liver.

By setting each of the ODEs equal to 0, we assume the model to be at steady state. We can then solve these ODEs to get expressions for the chemical concentration in each of the compartments

at steady state in terms of the exposure concentration, C_{H_2O}:

$$C_{brn,1} = \frac{\frac{F_{gill}}{P_{bw}}\left(\frac{F_{gill}}{P_{bw}} + F_{car}\right)(F_{liv} + k_{liv})P_{brn}C_{H_2O}}{F_{car}\left(F_{liv}k_{liv} + \frac{F_{gill}}{P_{bw}}(F_{liv} + k_{liv})\right)}$$

$$C_{liv,1} = \frac{\frac{F_{gill}}{P_{bw}}\left(\frac{F_{gill}}{P_{bw}} + F_{car}\right)F_{liv}P_{liv}C_{H_2O}}{F_{car}\left(F_{liv}k_{liv} + \frac{F_{gill}}{P_{bw}}(F_{liv} + k_{liv})\right)}$$

$$C_{oth,1} = \frac{\frac{F_{gill}}{P_{bw}}\left(\frac{F_{gill}}{P_{bw}} + F_{car}\right)(F_{liv} + k_{liv})P_{oth}C_{H_2O}}{F_{car}\left(F_{liv}k_{liv} + \frac{F_{gill}}{P_{bw}}(F_{liv} + k_{liv})\right)}$$

$$C_{art,1} = \frac{\frac{F_{gill}}{P_{bw}}\left(\frac{F_{gill}}{P_{bw}} + F_{car}\right)(F_{liv} + k_{liv})C_{H_2O}}{F_{car}\left(F_{liv}k_{liv} + \frac{F_{gill}}{P_{bw}}(F_{liv} + k_{liv})\right)}$$

$$C_{ven,1} = \frac{\frac{F_{gill}}{P_{bw}}\left((F_{brn} + F_{oth,1})(F_{liv} + k_{liv}) + (F_{liv})^2\right)C_{H_2O}}{F_{car}\left(F_{liv}k_{liv} + \frac{F_{gill}}{P_{bw}}(F_{liv} + k_{liv})\right)}$$

We can then define an expanded model in which the other tissues is split into a gonads compartment and a different other tissues:

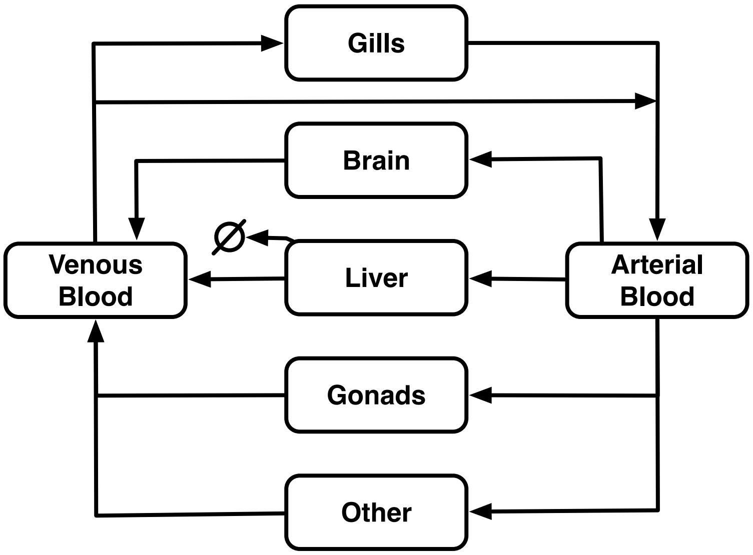

The system of ODEs describing the accumulation of chemical in the compartments are:

$$
\begin{aligned}
V_{brn}\frac{dC_{brn,2}}{dt} &= F_{brn}\left(C_{art,2} - \frac{C_{brn,2}}{P_{brn}}\right) \\
V_{liv}\frac{dC_{liv,2}}{dt} &= F_{liv}\left(C_{art,2} - \frac{C_{liv,2}}{P_{liv}}\right) - \frac{C_{liv,2}}{P_{liv}}k_{met} \\
V_{gon}\frac{dC_{gon}}{dt} &= F_{gon}\left(C_{art,2} - \frac{C_{gon}}{P_{gon}}\right) \\
V_{oth,2}\frac{dC_{oth,2}}{dt} &= F_{oth,2}\left(C_{art,2} - \frac{C_{oth,2}}{P_{oth,2}}\right) \\
V_{art}\frac{dC_{art,2}}{dt} &= \frac{F_{gill}}{P_{bw}}C_{H_2O} - F_{car}(C_{art,2} - C_{ven,2}) \\
V_{ven}\frac{dC_{ven,2}}{dt} &= -\frac{F_{gill}}{P_{bw}}C_{ven,2} - F_{car}C_{ven,2} + F_{brn}\frac{C_{brn,2}}{P_{brn}} + F_{liv}\frac{C_{liv,2}}{P_{liv}} + F_{oth,2}\frac{C_{oth,2}}{P_{oth}}
\end{aligned}
$$

Note that in this case $F_{car} = F_{brn} + F_{liv} + F_{gon} + F_{oth,2}$, so we can define $F_{oth,1} \equiv F_{oth,2} + F_{gon}$, assuming a constant total blood flow rate. Additionally, we can also define $V_{oth,1} \equiv V_{gon} + V_{oth,2}$, thus keeping the volume of the organism and the other compartments constant.

By setting each of the ODEs equal to 0, we can once again obtain expressions for the chemical concentration in each of the compartments at steady state in terms of the exposure concentration:

$$
\begin{aligned}
C_{brn,2} &= \frac{\frac{F_{gill}}{P_{bw}}\left(\frac{F_{gill}}{P_{bw}} + F_{car}\right)(F_{liv} + k_{liv})P_{brn}C_{H_2O}}{F_{car}\left(F_{liv}k_{liv} + \frac{F_{gill}}{P_{bw}}(F_{liv} + k_{liv})\right)} \\
C_{liv,2} &= \frac{\frac{F_{gill}}{P_{bw}}\left(\frac{F_{gill}}{P_{bw}} + F_{car}\right)F_{liv}P_{liv}C_{H_2O}}{F_{car}\left(F_{liv}k_{liv} + \frac{F_{gill}}{P_{bw}}(F_{liv} + k_{liv})\right)} \\
C_{gon} &= \frac{\frac{F_{gill}}{P_{bw}}\left(\frac{F_{gill}}{P_{bw}} + F_{car}\right)(F_{liv} + k_{liv})P_{gon}C_{H_2O}}{F_{car}\left(F_{liv}k_{liv} + \frac{F_{gill}}{P_{bw}}(F_{liv} + k_{liv})\right)} \\
C_{oth,2} &= \frac{\frac{F_{gill}}{P_{bw}}\left(\frac{F_{gill}}{P_{bw}} + F_{car}\right)(F_{liv} + k_{liv})P_{oth,2}C_{H_2O}}{F_{car}\left(F_{liv}k_{liv} + \frac{F_{gill}}{P_{bw}}(F_{liv} + k_{liv})\right)} \\
C_{art,2} &= \frac{\frac{F_{gill}}{P_{bw}}\left(\frac{F_{gill}}{P_{bw}} + F_{car}\right)(F_{liv} + k_{liv})C_{H_2O}}{F_{car}\left(F_{liv}k_{liv} + \frac{F_{gill}}{P_{bw}}(F_{liv} + k_{liv})\right)} \\
C_{ven,2} &= \frac{\frac{F_{gill}}{P_{bw}}\left((F_{brn} + F_{gon} + F_{oth,2})(F_{liv} + k_{liv}) + (F_{liv})^2\right)C_{H_2O}}{F_{car}\left(F_{liv}k_{liv} + \frac{F_{gill}}{P_{bw}}(F_{liv} + k_{liv})\right)}
\end{aligned}
$$

Just from direct comparison, we can see that the chemical concentrations in the brain, liver,

arterial blood and venous blood at steady state in both models are exactly the same. This indicates that the expansion of the single other compartment in the first model to two parallel compartments, gonads and other, in the second, each of which having the same incoming and outgoing connections, has no effect on the steady states of the other compartments.

Finally we can build one more model in which the number of compartments is the same as the first model, but the architecture is different in that the blood flowing out of the other tissue compartment enters the liver compartment rather than the venous blood:

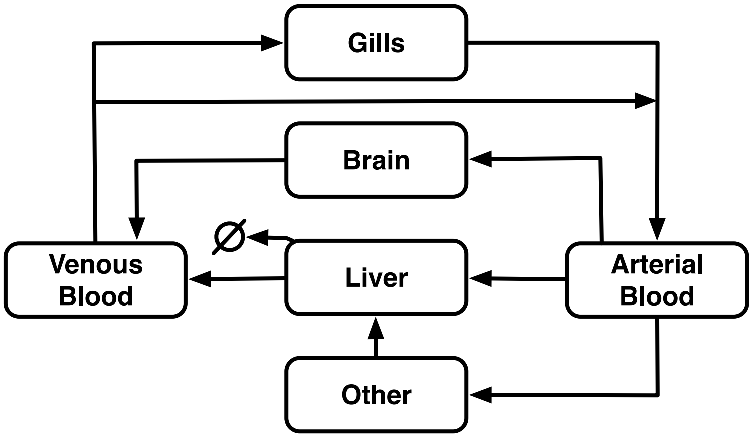

The system of ODEs describing the accumulation of chemical in the compartments are:

$$V_{brn}\frac{dC_{brn,3}}{dt} = F_{brn}\left(C_{art,3} - \frac{C_{brn,3}}{P_{brn}}\right)$$
$$V_{liv}\frac{dC_{liv,3}}{dt} = F_{liv}\left(C_{art,3} - \frac{C_{liv,3}}{P_{liv}}\right) + F_{oth,1}\left(\frac{C_{oth,3}}{P_{oth,1}} - \frac{C_{liv,3}}{P_{liv}}\right) - \frac{C_{liv,3}}{P_{liv}}k_{met}$$
$$V_{oth,1}\frac{dC_{oth,3}}{dt} = F_{oth,1}\left(C_{art,3} - \frac{C_{oth,3}}{P_{oth,1}}\right)$$
$$V_{art}\frac{dC_{art,3}}{dt} = \frac{F_{gill}}{P_{bw}}C_{H_2O} - F_{car}(C_{art,3} - C_{ven,3})$$
$$V_{ven}\frac{dC_{ven,3}}{dt} = -\frac{F_{gill}}{P_{bw}}C_{ven,3} - F_{car}C_{ven,3} + F_{brn}\frac{C_{brn,3}}{P_{brn}} + F_{liv}\frac{C_{liv,3}}{P_{liv}} + F_{oth,1}\frac{C_{liv,3}}{P_{liv}}$$

Note that because the other compartment in this model has the same incoming flow from the arterial blood, we maintained the same blood flow rate for it as in the first model ($F_{oth,1}$). Similarly, as the composition of the other tissue has not changed, the partition ratio remains the same ($P_{oth,1}$).

By setting each of the ODEs equal to 0, we can once again obtain expressions for the chemical

concentration in each of the compartments at steady state in terms of the exposure concentration:

$$C_{brn,3} = \frac{\frac{F_{gill}}{P_{bw}}\left(\frac{F_{gill}}{P_{bw}} + F_{car}\right)(F_{liv} + F_{oth,1} + k_{liv})P_{brn}C_{H_2O}}{F_{car}\left((F_{liv} + F_{oth,1})k_{liv} + \frac{F_{gill}}{P_{bw}}(F_{liv} + F_{oth,1} + k_{liv})\right)}$$

$$C_{liv,3} = \frac{\frac{F_{gill}}{P_{bw}}\left(\frac{F_{gill}}{P_{bw}} + F_{car}\right)(F_{liv} + F_{oth,1})P_{liv}C_{H_2O}}{F_{car}\left((F_{liv} + F_{oth,1})k_{liv} + \frac{F_{gill}}{P_{bw}}(F_{liv} + F_{oth,1} + k_{liv})\right)}$$

$$C_{oth,3} = \frac{\frac{F_{gill}}{P_{bw}}\left(\frac{F_{gill}}{P_{bw}} + F_{car}\right)(F_{liv} + F_{oth,1} + k_{liv})P_{oth,1}C_{H_2O}}{F_{car}\left((F_{liv} + F_{oth,1})k_{liv} + \frac{F_{gill}}{P_{bw}}(F_{liv} + F_{oth,1} + k_{liv})\right)}$$

$$C_{art,3} = \frac{\frac{F_{gill}}{P_{bw}}\left(\frac{F_{gill}}{P_{bw}} + F_{car}\right)(F_{liv} + F_{oth,1} + k_{liv})C_{H_2O}}{F_{car}\left((F_{liv} + F_{oth,1})k_{liv} + \frac{F_{gill}}{P_{bw}}(F_{liv} + F_{oth,1} + k_{liv})\right)}$$

$$C_{ven,3} = \frac{\frac{F_{gill}}{P_{bw}}\left(F_{brn}(F_{liv} + F_{oth,1} + k_{liv}) + (F_{liv} + F_{oth,1})^2\right)C_{H_2O}}{F_{car}\left((F_{liv} + F_{oth,1})k_{liv} + \frac{F_{gill}}{P_{bw}}(F_{liv} + F_{oth,1} + k_{liv})\right)}$$

From direct comparison we can see that the chemical concentrations in each of the compartments at steady state in this last model and the first model, while sharing a similar form, are ultimately different. Any instance of F_{liv} in the expressions for the first model are replaced with $F_{liv} + F_{oth,1}$ in the last model to account for the increased flow rate from the liver into the venous blood. The differences in the steady state chemical concentrations in each compartment are:

$$C_{brn,3} - C_{brn,1} = \frac{\frac{F_{gill}}{P_{bw}}(k_{liv})^2 F_{oth,1}\left(\frac{F_{gill}}{P_{bw}} + F_{car}\right)P_{brn}C_{H_2O}}{F_{car}\left(\frac{F_{gill}}{P_{bw}}(F_{liv} + k_{liv}) + F_{liv}k_{liv}\right)\left(\frac{F_{gill}}{P_{bw}}(F_{liv} + F_{oth,1} + k_{liv}) + (F_{liv} + F_{oth,1})k_{liv}\right)}$$

$$C_{liv,3} - C_{liv,1} = \frac{\left(\frac{F_{gill}}{P_{bw}}\right)^2 k_{liv}F_{oth,1}\left(\frac{F_{gill}}{P_{bw}} + F_{car}\right)P_{liv}C_{H_2O}}{F_{car}\left(\frac{F_{gill}}{P_{bw}}(F_{liv} + k_{liv}) + F_{liv}k_{liv}\right)\left(\frac{F_{gill}}{P_{bw}}(F_{liv} + F_{oth,1} + k_{liv}) + (F_{liv} + F_{oth,1})k_{liv}\right)}$$

$$C_{oth,3} - C_{oth,1} = \frac{\frac{F_{gill}}{P_{bw}}(k_{liv})^2 F_{oth,1}\left(\frac{F_{gill}}{P_{bw}} + F_{car}\right)P_{oth,1}C_{H_2O}}{F_{car}\left(\frac{F_{gill}}{P_{bw}}(F_{liv} + k_{liv}) + F_{liv}k_{liv}\right)\left(\frac{F_{gill}}{P_{bw}}(F_{liv} + F_{oth,1} + k_{liv}) + (F_{liv} + F_{oth,1})k_{liv}\right)}$$

$$C_{art,3} - C_{art,1} = \frac{\frac{F_{gill}}{P_{bw}}(k_{liv})^2 F_{oth,1}\left(\frac{F_{gill}}{P_{bw}} + F_{car}\right)C_{H_2O}}{F_{car}\left(\frac{F_{gill}}{P_{bw}}(F_{liv} + k_{liv}) + F_{liv}k_{liv}\right)\left(\frac{F_{gill}}{P_{bw}}(F_{liv} + F_{oth,1} + k_{liv}) + (F_{liv} + F_{oth,1})k_{liv}\right)}$$

$$C_{ven,3} - C_{ven,1} = \frac{\frac{F_{gill}}{P_{bw}}(k_{liv})^2 F_{oth,1}\left(\frac{F_{gill}}{P_{bw}} + F_{car}\right)C_{H_2O}}{F_{car}\left(\frac{F_{gill}}{P_{bw}}(F_{liv} + k_{liv}) + F_{liv}k_{liv}\right)\left(\frac{F_{gill}}{P_{bw}}(F_{liv} + F_{oth,1} + k_{liv}) + (F_{liv} + F_{oth,1})k_{liv}\right)}$$

We can show that each of these are positive, assuming a measurable, non-zero exposure

concentration, by first assuming the opposite:

$$
\begin{aligned}
C_{brn,3} - C_{brn,1} &\leq 0 \\
\frac{\frac{F_{gill}}{P_{bw}}(k_{liv})^2 F_{oth,1}\left(\frac{F_{gill}}{P_{bw}} + F_{car}\right) P_{brn} C_{H_2O}}{F_{car}\left(\frac{F_{gill}}{P_{bw}}(F_{liv} + k_{liv}) + F_{liv}k_{liv}\right)\left(\frac{F_{gill}}{P_{bw}}(F_{liv} + F_{oth,1} + k_{liv}) + (F_{liv} + F_{oth,1})k_{liv}\right)} &\leq 0 \\
\frac{F_{gill}}{P_{bw}}(k_{liv})^2 F_{oth,1}\left(\frac{F_{gill}}{P_{bw}} + F_{car}\right) P_{brn} C_{H_2O} &\leq 0 \\
C_{H_2O} &\leq 0
\end{aligned}
$$

$$
\begin{aligned}
C_{liv,3} - C_{liv,1} &\leq 0 \\
\frac{\left(\frac{F_{gill}}{P_{bw}}\right)^2 k_{liv} F_{oth,1}\left(\frac{F_{gill}}{P_{bw}} + F_{car}\right) P_{liv} C_{H_2O}}{F_{car}\left(\frac{F_{gill}}{P_{bw}}(F_{liv} + k_{liv}) + F_{liv}k_{liv}\right)\left(\frac{F_{gill}}{P_{bw}}(F_{liv} + F_{oth,1} + k_{liv}) + (F_{liv} + F_{oth,1})k_{liv}\right)} &\leq 0 \\
\left(\frac{F_{gill}}{P_{bw}}\right)^2 k_{liv} F_{oth,1}\left(\frac{F_{gill}}{P_{bw}} + F_{car}\right) P_{liv} C_{H_2O} &\leq 0 \\
C_{H_2O} &\leq 0
\end{aligned}
$$

$$
\begin{aligned}
C_{oth,3} - C_{oth,1} &\leq 0 \\
\frac{\frac{F_{gill}}{P_{bw}}(k_{liv})^2 F_{oth,1}\left(\frac{F_{gill}}{P_{bw}} + F_{car}\right) P_{oth,1} C_{H_2O}}{F_{car}\left(\frac{F_{gill}}{P_{bw}}(F_{liv} + k_{liv}) + F_{liv}k_{liv}\right)\left(\frac{F_{gill}}{P_{bw}}(F_{liv} + F_{oth,1} + k_{liv}) + (F_{liv} + F_{oth,1})k_{liv}\right)} &\leq 0 \\
\frac{F_{gill}}{P_{bw}}(k_{liv})^2 F_{oth,1}\left(\frac{F_{gill}}{P_{bw}} + F_{car}\right) P_{oth,1} C_{H_2O} &\leq 0 \\
C_{H_2O} &\leq 0
\end{aligned}
$$

$$
\begin{aligned}
C_{art,3} - C_{art,1} &\leq 0 \\
\frac{\frac{F_{gill}}{P_{bw}}(k_{liv})^2 F_{oth,1}\left(\frac{F_{gill}}{P_{bw}} + F_{car}\right) C_{H_2O}}{F_{car}\left(\frac{F_{gill}}{P_{bw}}(F_{liv} + k_{liv}) + F_{liv}k_{liv}\right)\left(\frac{F_{gill}}{P_{bw}}(F_{liv} + F_{oth,1} + k_{liv}) + (F_{liv} + F_{oth,1})k_{liv}\right)} &\leq 0 \\
\frac{F_{gill}}{P_{bw}}(k_{liv})^2 F_{oth,1}\left(\frac{F_{gill}}{P_{bw}} + F_{car}\right) C_{H_2O} &\leq 0 \\
C_{H_2O} &\leq 0
\end{aligned}
$$

$$C_{ven,3} - C_{ven,1} \leq 0$$

$$\frac{\frac{F_{gill}}{P_{bw}}(k_{liv})^2 F_{oth,1}\left(\frac{F_{gill}}{P_{bw}} + F_{car}\right) C_{H_2O}}{F_{car}\left(\frac{F_{gill}}{P_{bw}}(F_{liv} + k_{liv}) + F_{liv}k_{liv}\right)\left(\frac{F_{gill}}{P_{bw}}(F_{liv} + F_{oth,1} + k_{liv}) + (F_{liv} + F_{oth,1})k_{liv}\right)} \leq 0$$

$$\frac{F_{gill}}{P_{bw}}(k_{liv})^2 F_{oth,1}\left(\frac{F_{gill}}{P_{bw}} + F_{car}\right) C_{H_2O} \leq 0$$

$$C_{H_2O} \leq 0$$

Since we are assuming a positive value for C_{H_2O}, we can see that each of these are false. We can then accept that the difference $C_{i,3} - C_{i,1}$ for $i = art, ven, brn, liv, oth$ is always positive. Additionally, we can assume that the full body concentration of the last model ($C_{tot,3} \equiv C_{art,3} + C_{ven,3} + C_{brn,3} + C_{liv,3} + C_{oth,3}$) would then be larger than the full body concentration of the first model ($C_{tot,1} \equiv C_{art,1} + C_{ven,1} + C_{brn,1} + C_{liv,1} + C_{oth,1}$). This comparison then indicates that the differences in connectivity of the distinct compartments within a model can then influence the steady state full body chemical concentration of the model and the predicted exposure concentration of the associated reverse toxicokinetic model.

8 Approximation of the departure of in vitro data from control levels

We can approximate the concentration where the *in vitro* assay activity departs from control levels. In this section, we will investigate that point using the model:

$$y = \frac{(x/k)^h}{1 + (x/k)^h}. \tag{S44}$$

To do this we will simplify the sigmoidal curve we get when plotting Eq. S44 on a semi-log plot (Fig. S10) to a piecewise linear function. The upper and lower horizontal asymptotes provide two parts of this piecewise linear function, estimating y when $x \ll k$ and $x \gg k$. To derive a linear expression for y when x is close to k, we must first find the inflection point. To do this, we take the derivative of y with respect to $\log x$:

$$\frac{dy}{d\log x} = \frac{h(x/k)^h}{(1 + (x/k)^h)^2}. \tag{S45}$$

Then we can find the second derivative of y with respect to $\log x$:

$$\frac{d^2y}{d\log x^2} = \frac{-h^2(x/k)^h(-1 + (x/k)^h)}{(1 + (x/k)^h)^3} \tag{S46}$$

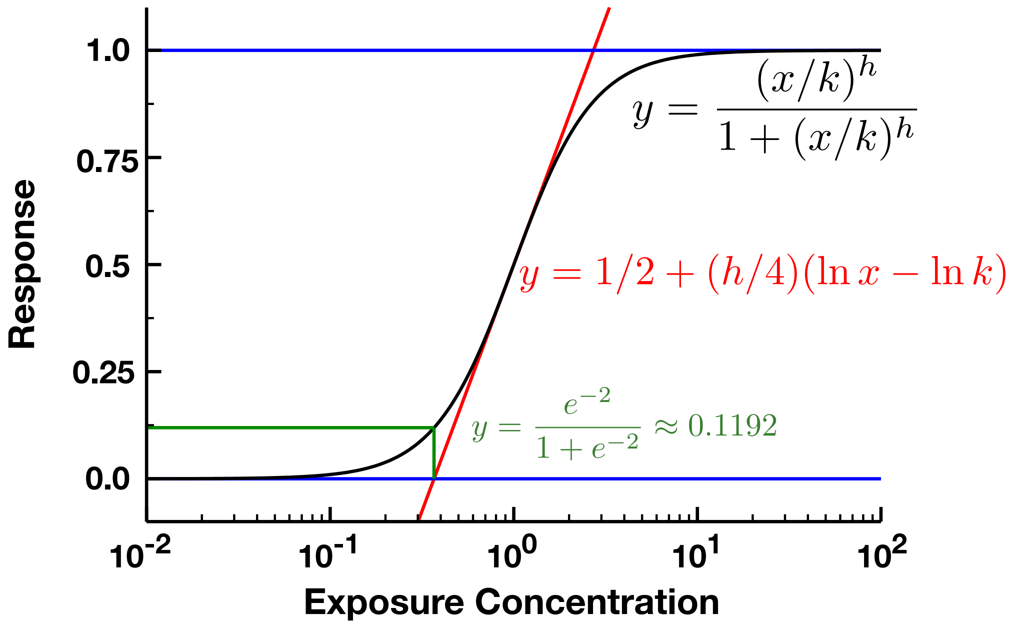

Figure S10: The response of a system to increasing exposure concentrations. Note that the x-axis has a logarithmic scale. $k = 1$ and $h = 2$

We can then set Eq. S46 to 0:

$$\begin{aligned} \frac{-h^2(x/k)^h(-1+(x/k)^h)}{(1+(x/k)^h)^3} &= 0 \\ -h^2(x/k)^h(-1+(x/k)^h) &= 0 \\ -1+(x/k)^h &= 0 \\ x &= k \end{aligned} \tag{S47}$$

Thus the inflection point of Eq. S44 is at $x = k$, assuming $x > 0$. We can then find the line tangent to the inflection point by first finding the slope at this point:

$$\begin{aligned} \left(\frac{dy}{d\ln x}\right)(x=k) &= \frac{h(k/k)^h}{(1+(k/k)^h)^2} \\ &= \frac{h}{4} \end{aligned}$$

This gives us the equation:

$$y = \frac{h}{4}\ln x + b \tag{S48}$$

We can then find the value of y at $x = k$:

$$y(x = k) = \frac{(k/k)^h}{1 + (k/k)^h} = \frac{1}{2}$$

We then substitute these values for x and y into S48:

$$\frac{1}{2} = \frac{h}{4} \ln k + b$$
$$b = \frac{1}{2} - \frac{h}{4} \ln k$$

Finally giving us the equation:

$$y = \frac{h}{4}(\ln x - \ln k) + \frac{1}{2} \tag{S49}$$

This line and the horizontal asymptotes are plotted in red and blue, respectively, in Fig. S10. We can then find the exposure concentration where the lower horizontal asymptote and the linear approximation of the power-law regime of the model response intersect:

$$0 = \frac{h}{4}(\ln x - \ln k) + \frac{1}{2}$$
$$-\frac{2}{h} = \ln x - \ln k$$
$$\ln x = \ln k - \frac{2}{h}$$
$$x = e^{\ln k - 2/h}$$
$$x = ke^{-2/h}$$

We can then find the value of our original model at this exposure concentration:

$$y = \frac{(e^{-2/h})^h}{1 + (e^{-2/h})^h} = \frac{1}{1 + e^2} \approx 0.1192$$

Thus showing that the point at which the model departs from the control levels occurs when the response is about 12% of its shift from basal to maximal response. This crossover point is independent of any parameter values and is therefore universal for all curves that can be fit to a sigmoid function. As such, values measuring 10% toxicity (e.g., LC10, EC10) are a good approximation of this cross-over point.

References

D'Souza et al., 1987. D'Souza, R., Francis, W., Bruce, R. and Andersen, M. (1987). Physiologically Based Pharmacokinetic Model for Ethylene Dichloride and Its Application in Risk Assessment. In Drinking Water and Health, Volume 8: Pharmacokinetics in Risk Assessment pp. 286–311. National Academies Press.

Hansch et al., 1995. Hansch, C., Leo, A. and Hoekman, D. (1995). Exploring QSAR: Hydrophobis, electronic, and steric constants, vol. 2,. American Chemical Society.

Li et al., 2011. Li, Z., Kroll, K., Jensen, K., Villeneuve, D., Ankley, G., Brian, J., Sepulveda, M., Orlando, E., Lazorchak, J., Kostich, M., Armstrong, B., Denslow, N. and Watanabe, K. (2011). A computational model of the hypothalamic: pituitary: gonadal axis in female fathead minnows (Pimephales promelas) exposed to 17alpha-ethynylestradiol and 17beta-trenbolone. BMC Syst Biol *5*, 63.

Péry et al., 2013. Péry, A. R., Devillers, J., Brochot, C., Mombelli, E., Palluel, O., Piccini, B., Brion, F. and Beaudouin, R. (2013). A Physiologically Based Toxicokinetic Model for the Zebrafish Danio rerio. Environmental Science & Technology *48*, 781–790.

Peters, 2012. Peters, S. A. (2012). Physiologically-Based Pharmacokinetic (PBPK) Modeling and Simulations. John Wiley & Sons, Hoboken, NJ.

R Core Team, 2015. R Core Team (2015). R: A Language and Environment for Statistical Computing. R Foundation for Statistical Computing Vienna, Austria.
